# Multi-omics-based insights into tomato adaptation to multifactorial stress combination

**DOI:** 10.1093/plphys/kiaf519

**Published:** 2025-10-14

**Authors:** Lidia S Pascual, Enrique Serna, Abdul Ghani, Zhen Lyu, Manish Sridhar Immadi, Trupti Joshi, Mohit Verma, José L Rambla, Aurelio Gómez-Cadenas, Ron Mittler, Sara I Zandalinas

**Affiliations:** Department of Biology, Biochemistry and Environmental Sciences, University Jaume I, Castelló de la Plana 12071, Spain; Department of Biology, Biochemistry and Environmental Sciences, University Jaume I, Castelló de la Plana 12071, Spain; Department of Biomedical Informatics, Biostatistics and Medical Epidemiology, University of Missouri, Columbia, MO 65211, USA; Department of Electrical Engineering and Computer Science, University of Missouri, Columbia, MO 65211, USA; Department of Biomedical Informatics, Biostatistics and Medical Epidemiology, University of Missouri, Columbia, MO 65211, USA; Department of Biomedical Informatics, Biostatistics and Medical Epidemiology, University of Missouri, Columbia, MO 65211, USA; Department of Electrical Engineering and Computer Science, University of Missouri, Columbia, MO 65211, USA; Christopher S. Bond Life Sciences Center, University of Missouri, Columbia, MO 65211, USA; MU Institute for Data Science and Informatics, University of Missouri, Columbia, MO 65211, USA; Department of Biomedical Informatics, Biostatistics and Medical Epidemiology, University of Missouri, Columbia, MO 65211, USA; Department of Biology, Biochemistry and Environmental Sciences, University Jaume I, Castelló de la Plana 12071, Spain; Department of Biology, Biochemistry and Environmental Sciences, University Jaume I, Castelló de la Plana 12071, Spain; Christopher S. Bond Life Sciences Center, University of Missouri, Columbia, MO 65211, USA; Division of Plant Science and Technology, College of Agriculture Food and Natural Resources, University of Missouri, Columbia, MO 65211, USA; Department of Biology, Biochemistry and Environmental Sciences, University Jaume I, Castelló de la Plana 12071, Spain

## Abstract

Multifactorial stress combination (MFSC) is emerging as a major constraint to crop productivity under different climate change scenarios. While the physiological impacts of MFSC have been previously characterized in different plant species, the molecular and metabolic effects of MFSC remain poorly defined. Here, we used an integrative multi-omics approach to dissect the response of tomato (*Solanum lycopersicum*) plants to an MFSC of up to 6 low-intensity abiotic stressors. Our analysis uncovered a complexity-dependent molecular program in tomato. Transcriptomic analysis identified a core set of 194 transcripts commonly altered across all stress conditions, along with 155 transcription factors (TFs) specifically regulated under high-complexity conditions (4-, 5-, and 6-stress combinations). Focusing on heat-associated MFSC responses, we identified 103 transcripts uniquely responsive to these conditions, including 2 TFs (Zinc finger TF 32 and a B3 family protein) that may act as master regulators of all heat-associated MFSCs. Metabolomic profiling revealed a pronounced reprogramming of primary metabolism under MFSC, marked by decreased levels of tricarboxylic acid intermediates and accumulation of sugars, γ-aminobutyric acid, and branched-chain amino acids, suggesting a trade-off that favors osmoprotection and redox homeostasis over energy-intensive processes. Comparative analyses across tomato, Arabidopsis, Chlamydomonas, rice, and soybean highlighted a conserved molecular signature associated with MFSC. Integrated omics correlation analysis uncovered functional links among phytohormone signaling, photosynthetic efficiency, and key MFSC-related transcripts and metabolic hubs. Together, we reveal a coordinated and complexity-dependent molecular program in tomato, offering insights into plant adaptation to MFSC and identifying candidate regulatory and metabolic markers for engineering climate-resilient crops.

## Teaser

Multifactorial stress combination triggers a complexity-dependent molecular and metabolic reprogramming.

## Introduction

Climate change is altering environmental conditions, leading to more frequent and severe weather fluctuations across different regions of the world (Mazdiyasni and AghaKouchak 2015; Kossin et al. 2020; Zandalinas et al. 2021a; IPCC 2022; Rivero et al. 2022). These changes are subjecting crops to unprecedented levels of abiotic stresses, including extreme temperatures, prolonged droughts, irregular precipitation patterns, increased soil salinity, and nutrient imbalances. Adding to these challenges is the widespread presence of environmental pollutants, including heavy metals, microplastics, persistent organic pollutants, ozone, and agrochemicals, which further alter plant stress responses by affecting key physiological and metabolic processes (Maddela et al. 2022; Xing et al. 2023). In many cases, these stresses do not occur in isolation but rather in combination, either simultaneously or sequentially, creating complex “stress combination” environmental challenges for plants (Mittler 2006; Mittler and Blumwald 2010; Bailey-Serres et al. 2019; Zandalinas et al. 2021a, 2024; Pascual et al. 2022; Zandalinas and Mittler 2022). Different stress combinations were reported to have severe consequences for global food production, as they impair plant growth, reduce yields, and disrupt key physiological and reproductive processes (Prasch and Sonnewald 2013; Zandalinas et al. 2016; Shaar-Moshe et al. 2017; Zsögön et al. 2022; Rivero et al. 2022; Peláez-Vico et al. 2024; Zagorščak et al. 2025). Complex conditions of stress combination have been recently termed multifactorial stress combination (MFSC), a condition in which plants face multiple abiotic and/or biotic stresses simultaneously, leading to cumulative and often unpredictable negative impacts on growth, metabolism, and survival (Zandalinas et al. 2021a,b, 2024; Pascual et al. 2023; Bi et al. 2024; Peláez-Vico et al. 2024; Sinha et al. 2024). Unlike single-stress conditions, MFSC presents a unique challenge to plants, disrupting homeostasis at multiple levels, affecting gene expression, cellular metabolism, and signaling networks in ways that are different from individual stress responses (Zandalinas et al. 2021a, 2024; Pascual et al. 2022, 2023; Zandalinas and Mittler 2022; Bi et al. 2024; Peláez-Vico et al. 2024; Sinha et al. 2024).

A previous study in *Arabidopsis thaliana* showed that plants subjected to MFSC display unique transcriptomic signatures, indicating a reprogramming of stress-response pathways that markedly differed from those observed under single-stress conditions or simple combinations of 2-factor stresses (Zandalinas et al. 2021b). These findings highlighted the complexity of plant adaptation to multifactorial stress and suggested that responses at the transcriptome level are intricately linked to stress perception, signaling, and metabolic reconfiguration. In major crop species, such as rice (*Oryza sativa*), maize (*Zea mays*), soybean (*Glycine max*), and tomato (*Solanum lycopersicum*), exposure to MFSC has been associated with reductions in biomass, altered phytohormone signaling, and disruptions in key physiological processes essential for growth and reproduction (Pascual et al. 2023; Peláez-Vico et al. 2024; Sinha et al. 2024). Proteomic analysis in rice has further demonstrated that each unique MFSC condition triggers a distinct molecular response, reinforcing the idea that plants employ highly specific regulatory mechanisms to cope with complex environmental stressors (Sinha et al. 2024). Similarly, studies in soybean plants subjected to MFSC demonstrated that each stress combination condition triggered a distinct transcriptomic response, with remarkable differences between reproductive (flowers) and vegetative (leaves) tissues, and that key genes linked to vegetative, physiological, and reproductive processes were differentially regulated under MFSC (Peláez-Vico et al. 2024). These findings suggest that plant responses to MFSC are not only species-specific but also organ-specific, requiring finely tuned regulatory mechanisms to optimize survival and reproduction under multiple stress conditions.

While the studies described above provided crucial insights into how plants respond to MFSC at the transcriptomic and proteomic levels, a comprehensive multi-omics investigation integrating metabolomic and transcriptomic responses in tomato remains limited. Given the central role of metabolites in stress adaptation, examining how metabolic networks are remodeled in response to MFSC, as well as identifying the transcriptomic changes associated with this response, is essential for understanding the full scope of plant responses under complex environmental conditions. Metabolites serve as key intermediates in stress signaling, energy balance, and protective mechanisms (e.g. Cai et al. 2020; Itam et al. 2020; Carrera et al. 2021; Wu et al. 2023; Pardo-Hernández et al. 2024), making their analysis a crucial complement to multi-omics studies aimed at unraveling the molecular basis of stress adaptation. In this study, we used an integrative multi-omics approach to investigate the transcriptomic and metabolomic responses of tomato plants to MFSC. By combining large-scale transcriptome and metabolome profiling, we aimed at constructing a comprehensive resource that provides a detailed molecular map of tomato responses to complex environmental challenges. The integration of hormonal and physiological data with transcriptomics and metabolomics further uncovered interactions that connect hormone signaling, photosynthetic efficiency, and key transcripts and metabolites associated with MFSC in tomato plants. We hope that the datasets and analyses presented in this study will serve as a valuable reference for future research on crop adaptation to MFSC, as well as aid in the development of resilient crops with heightened tolerance to different global environmental change scenarios.

## Results

### Transcriptomic reprogramming in tomato plants subjected to MFSC

**Figure 1.**
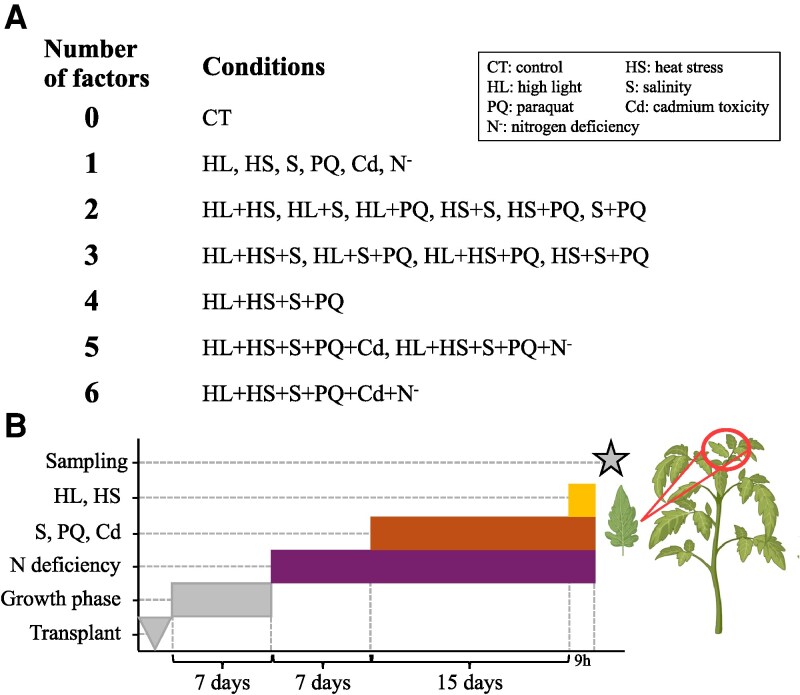
The experimental design used to study the transcriptomic and metabolomic responses of tomato plants to MFSC. **A)** The stresses/stress combinations used in the study. N^−^, HS, S, HL, Cd, and PQ were applied up to a combination of all 6 factors. HL, HS, S, and PQ were combined in all possible combinations of 2-, 3-, and 4-factor, and Cd and N^−^ were added to generate 5-and 6-factor MFSCs. **B)** Schematic representation of the timeline used to subject tomato plants to MFSC. The figure shows the tissues sampled from tomato plants for the transcriptomic and metabolomic analyses.

We previously investigated how a MFSC of up to 6 different abiotic factors affected tomato growth, physiology, and hormonal reprogramming (Pascual et al. 2023). That study used a MFSC experimental setup combining high light (HL), heat stress (HS), salinity (S), the herbicide paraquat (PQ), the heavy metal cadmium (Cd), and nitrogen deficiency (N^−^) as shown in Fig. 1. N^−^ was induced 1 wk post-transplanting by supplying a modified Hoagland solution with reduced nitrogen content (10% of N content). Subsequently, plants were subjected to additional abiotic stressors, including S (75 mm NaCl), PQ (1 *μ*M PQ), and Cd (10 *μ*M CdSO_4_), either individually or in all possible combinations for 15 d. Finally, control (CT) and stressed plants were subjected to HL (700 *μ*mol m^−2^s^−1^) and/or HS (37 °C) for 9 h, as shown in Fig. 1 and described by Pascual et al. (2023).

**Figure 2.**
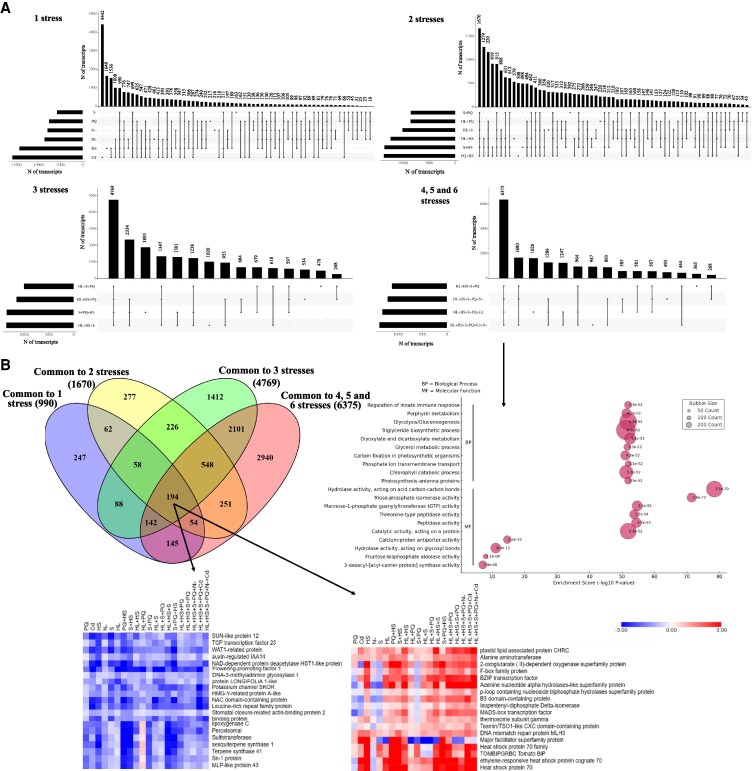
The transcriptomic response of tomato plants to MFSC. **A)** UpSet plots showing the overlap between transcripts significantly altered in their expression in tomato plants in response to: individual stresses (1 stress), combinations of 2 stresses (2 stresses), combinations of 3 stresses (3 stresses), and combinations of 4, 5, and 6 stresses (4, 5, and 6 stresses). A GO enrichment analysis of differentially expressed transcripts common to 4, 5-, and 6-stress combinations (from A, common 6,375 transcripts) is shown below (arrow). **B)** Venn diagram displaying common MFSC-response transcripts across all stress conditions. Heat maps depicting the expression of selected common transcripts with a general downregulation (left) or upregulation (right) pattern across all stress conditions (from B, common 194 transcripts; arrow) are shown below. A complete heat map is shown in Supplementary Fig. S6.

To dissect the molecular response to MFSC, an RNA-seq analysis of tomato plants subjected to the different stress combinations described by Pascual et al. (2023) was performed (Supplementary Tables S1 to S20, Supplementary Fig. S1). UpSet plots (Fig. 2A) revealed that Cd treatment had the most robust effect on transcriptomic responses among individual stress conditions, with over 4,440 transcripts specifically altered compared to CT. In addition, 990 transcripts were common to all individual stresses (Fig. 2A, Supplementary Table S21). When combining 2 stresses, more than 1,600 transcripts showed significant changes across all 2-factor treatments (Fig. 2A, Supplementary Table S22). Similarly, 4,769 transcripts were commonly affected across all 3-factor stress combinations (Fig. 2A, Supplementary Table S23), and 6,375 transcripts were altered under all MFSC involving 4, 5, and 6 stresses combined (Fig. 2A, Supplementary Table S24). These findings revealed a high degree of transcript overlap between different stress combinations and suggested that a conserved core of stress-response transcripts could participate in the response of tomato to MFSC. A gene ontology (GO) enrichment analysis of the 6,375 commonly altered transcripts among 4-, 5-, and 6-stress combinations revealed that these transcripts were associated with different metabolic pathways, including glycolysis/gluconeogenesis, carbon fixation and glyoxylate and dicarboxylate metabolism, and chlorophyll catabolism (Fig. 2A, bottom). In terms of subcellular localization, these encoded proteins were predominantly localized to the nucleus, chloroplast, and cytoplasm, followed by the mitochondria and plasma membrane. This distribution reflects the potential involvement of key regulatory hubs (nucleus), energy metabolism and photosynthesis-related processes (chloroplast and mitochondria), primary metabolic functions (cytoplasm), and stress signaling/transport mechanisms (plasma membrane) in the MFSC response of tomato plants (Supplementary Fig. S2). In agreement with earlier findings in *A. thaliana* (Zandalinas et al. 2021b) and soybean (Peláez-Vico et al. 2024), in which MFSC resulted in unique transcriptomic profiles that differed from single- or 2-factor stress response, our data included a considerable number of different MFSC unique transcripts that were altered in their expression in response to each stress combination (Fig. 2A).

Several heat shock proteins (HSPs) including members of the small HSP20 family (Solyc01g102960, Solyc03g113930, Solyc05g014280, Solyc08g062437), HSP70 (Solyc01g099660), and HSP90-1 (Solyc03g007890) were markedly upregulated under conditions of MFSC, while others showed different expression patterns depending on the stress/stress combination applied (e.g. Solyc02g080410, Solyc02g080470, or Solyc03g113180; Supplementary Fig. S3). In addition, transcripts involved in reactive oxygen species (ROS) metabolism (production and scavenging), critical for maintaining redox homeostasis and signaling during stress adaptation (Mittler 2017; Zandalinas et al. 2021b), displayed distinct expression patterns depending on the specific stress combinations applied. For example, ferredoxin Solyc01g103920, a key component in electron transport and ROS modulation (Moreau et al. 2020), was exclusively upregulated in all stress combinations that included HL, pointing to a light-driven redox response. In contrast, specific aldehyde oxidases (Solyc01g088200 and Solyc11g071610), enzymes implicated in both ROS metabolism and hormone biosynthesis (Devireddy et al. 2021), were only induced under HL + HS + S + PQ + N^−^ and HS, respectively, indicating a tailored transcriptional activation linked to oxidative stress severity and type (Supplementary Fig. S4). Analysis of the expression pattern of transcripts related to ROS scavenging (Supplementary Fig. S5) showed a clear activation of antioxidant defense mechanisms under MFSC. Several key enzymes associated with ROS detoxification were upregulated under these conditions, including peroxidase 40 (Solyc01g058520), ascorbate peroxidase (Solyc09g007270), catalase (Solyc01g100640, Solyc04g082460), Cu/Zn superoxide dismutase (Solyc03g062890), and Fe superoxide dismutase (Solyc03g095180). Notably, the expression of transcripts encoding the peroxidase 40 was upregulated in response to HS and all combinations involving HS, highlighting its central role in heat-associated oxidative stress responses. In contrast, several glutaredoxins exhibited stress condition-specific upregulation, being induced only under particular stresses/stress combinations (Supplementary Fig. S5). The expression patterns described above underscore the highly tailored and context-dependent nature of the transcriptomic response of tomato to complex environmental challenges, further supporting the notion that MFSC elicits unique regulatory programs distinct from those triggered by individual or 2-stress exposures.

Further analysis of transcripts altered across all stress conditions revealed that 194 transcripts were significantly upregulated or downregulated under all stress treatments (Fig. 2B; Supplementary Table S25). Among them, transcripts encoding Flowering-promoting factor 1 (Solyc01g066980), Auxin-regulated IAA14 (Solyc09g083290), and the transcription factor (TF) TCP23 (Solyc05g007420) were downregulated in response to all stresses (Fig. 2B, left, Supplementary Fig. S6, and Supplementary Table S25), whereas other transcripts, including those encoding alanine aminotransferase (Solyc03g123600), DNA mismatch repair protein MLH3 (Solyc02g082633), and plastid lipid associated protein CHRC (Solyc02g081170) were upregulated under all stress conditions (Fig. 2B, right, Supplementary Fig. S6, and Supplementary Table S25). These findings suggest that plants exposed to MFSC undergo extensive transcriptional remodeling to mitigate cumulative stress impacts, with specific pathways being preferentially activated or suppressed depending on stress complexity. The complexity-dependent nature of tomato transcriptional responses to MFSC was further supported by a principal component analysis (PCA), which showed tight clustering of control and single-stress samples, in contrast to the broad dispersion of MFSC conditions (Supplementary Fig. S1). Notably, MFSC treatments involving heat stress followed a distinct pattern, highlighting the strong influence of heat on the transcriptional reconfiguration of tomato under the different stress combinations.

### Expression of transcription factors in tomato plants subjected to MFSC

**Figure 3.**
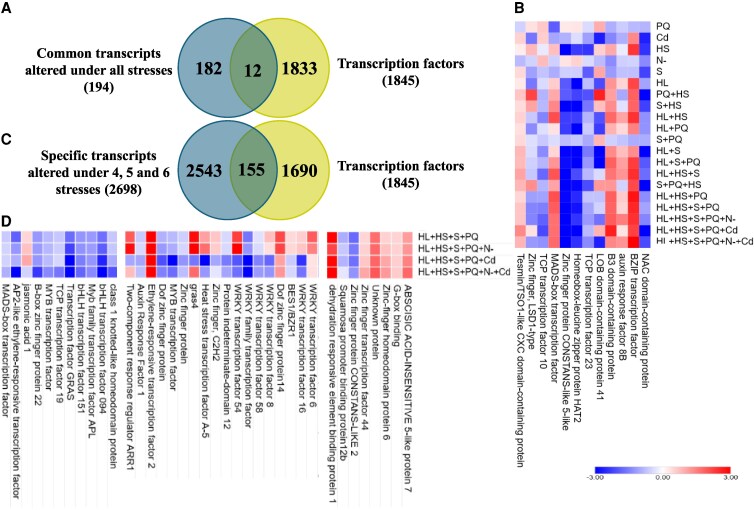
Expression of TFs associated with MFSC in tomato plants. **A)** Venn diagram showing the number of TFs found among the 194 common MFSC transcripts (from Fig. 2B). **B)** Heat map displaying the expression patterns of the 12 TFs identified in A under each stress/stress combination. **C)** Venn diagram showing the number of TFs specifically altered in response to 4-, 5-, and 6-stress combinations (not altered under other conditions). **D)** Partial heat map of the expression pattern of selected commonly altered TFs from C (full heat map is shown in Supplementary Fig. S7). Transcription factors with a general downregulation pattern in response to 4-, 5-, and 6-stress combinations are shown on the left; transcription factors with a contrasting expression pattern across 4-, 5-, and 6-stress combinations are shown in the middle; transcription factors with a general upregulation pattern in response to 4-, 5-, and 6-stress combinations are shown on the right.

To identify key TFs involved in the MFSC response of tomato plants, we analyzed clustered expression patterns of TF-encoding transcripts across all different stress combinations (Fig. 3A, Supplementary Table S26). This analysis revealed a small subset of TFs (12) that were commonly altered in response to all stress conditions, suggesting the existence of core regulatory elements governing general stress adaptation in tomato plants. Among these TFs, some showed a general repression expression pattern in response to the different stress conditions, such as NAC domain-containing protein (Solyc01g104900), TCP TF 23 (Solyc05g007420), HAT2 (Solyc06g060830), or CONSTANS-like zinc finger protein (Solyc07g006630), while others showed an upregulation expression pattern in response to stress, including BZIP TF (Solyc01g109880) or MADS-box TF (Solyc07g052700) (Fig. 3B).

To further explore the transcriptional response of tomato to MFSC, we determined the overlap between all tomato TFs and transcripts altered specifically under 4-, 5-, and 6-stress conditions (not altered under any other condition; 2,698 transcripts; Supplementary Table S27). As shown in Fig. 3C, Supplementary Fig. S7, and Supplementary Table S28, a total of 155 TFs were exclusively altered in these high-complex stress scenarios (i.e. MFSC). Interestingly, TFs belonging to the WRKY, NAC, and MYB families were significantly enriched in the group of TFs exclusively altered in response to 4-, 5-, and 6-stress combinations (Fig. 3D, Supplementary Fig. S7). This pattern suggests that these TFs could become increasingly important as stress complexity levels increase. The presence of abscisic acid (ABA)-responsive element binding TFs (AREB/ABF TFs), which operate through ABA-dependent signaling pathways (Yoshida et al. 2015), along with ethylene-responsive TFs (ERFs; Qi et al. 2023), suggests that ABA and ethylene signaling pathways are likely key regulators of plant responses to MFSC (Fig. 3D, Supplementary Fig. S7). In addition, promoter analysis of the genes encoding the 2,698 transcripts, specifically altered under 4-, 5-, and 6-stress conditions, revealed that a substantial number of these promoters contained binding sites for WRKY, NAC, MYB, heat shock factor (HSF), and/or ERF (Supplementary Table S29). These findings provide further support for the regulatory relevance of these TF families in orchestrating plant responses to MFSC.

### Common responses to MFSC between unicellular and multicellular photosynthetic organisms

**Figure 4.**
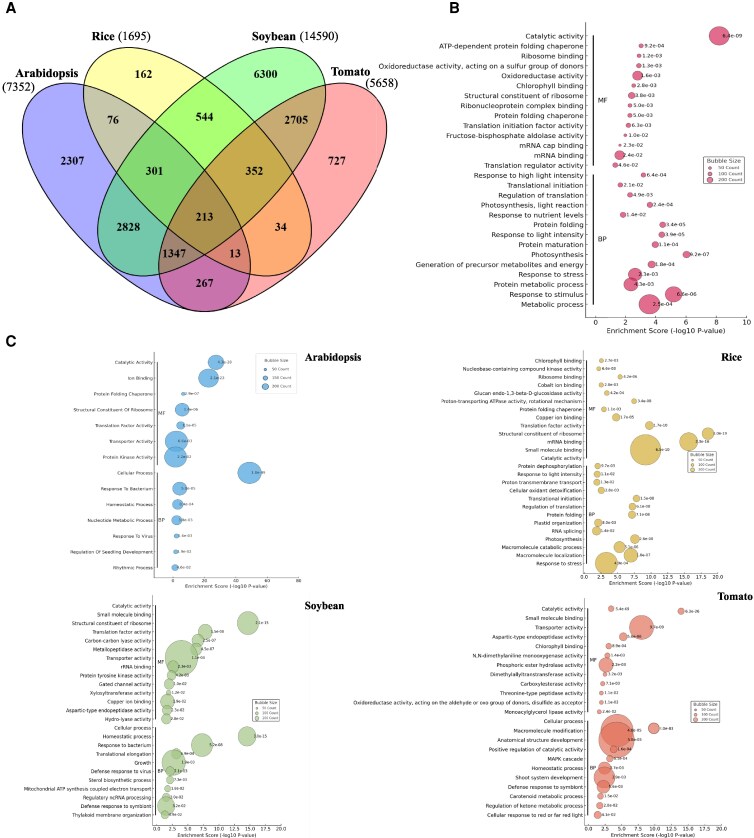
Molecular responses to MFSC in multicellular (tomato, soybean, rice, and Arabidopsis) organisms. **A)** Venn diagram comparing the molecular responses to MFSC of different plant species: tomato (transcripts significantly altered under 4-, 5-, and 6-stress combinations; this study), soybean (transcripts significantly altered under 4- and 5-stress combinations; Peláez-Vico et al. 2024), rice (proteins significantly altered under 4- and 5-stress combinations; Sinha et al. 2024), and Arabidopsis (transcripts significantly altered under 4-, 5-, and 6-stress combination; Zandalinas et al. 2021b). **B)** GO analysis of the 213 genes commonly altered in tomato, Arabidopsis, soybean, and rice in response to MFSC (from A). **C)** GO analysis of transcripts that are specifically altered in each species under MFSC (From A: 2,307 in Arabidopsis, 162 in rice, 6,300 in soybean, and 727 in tomato).

Beyond tomato, recent studies examined the molecular responses to MFSC of major crops and model organisms (Zandalinas et al. 2021b; Peláez-Vico et al. 2024; Sinha et al. 2024). The availability of transcriptomic and proteomic MFSC datasets from different plant species allowed us to compare their molecular responses to MFSC. To perform this correlation, *A. thaliana* homologs were identified for MFSC-responsive transcripts in soybean and tomato, as well as for proteins in rice, using the OMA Browser genome pair orthology tool (Altenhoff et al. 2021; Supplementary Tables S30 to S35). We then conducted a cross-species comparison among transcripts responsive to 4-, 5-, and 6-stress conditions in tomato (this study) and *A. thaliana* (Zandalinas et al. 2021b), transcripts responsive to 4- and 5-stress conditions in soybean (Peláez-Vico et al. 2024), along with proteins that responded to 4- and 5-stress conditions in rice (Sinha et al. 2024). This analysis revealed a shared subset of 213 genes commonly altered in their expression across these major multicellular organisms (Fig. 4A, Supplementary Table S36). These genes were predominantly involved in responses to stimulus/stress, translation, protein folding, and photosynthetic processes (Fig. 4B), indicating the presence of a conserved transcriptional core associated with plant responses to increasing environmental complexity. Subcellular localization analysis further showed that the proteins encoded by these conserved transcripts were mainly associated with the cytoplasm, as well as with other key compartments involved in photosynthetic processes, highlighting the relevance of energy metabolism in the common mechanisms that plants employ to cope with MFSC (Supplementary Fig. S8A). To better understand species-specific responses to MFSC, a GO enrichment analysis of transcripts uniquely altered in each species, was performed (Fig. 4C). While some functional categories were commonly enriched, such as transporter and catalytic activities, essential for nutrient redistribution and metabolic adjustment, distinct biological priorities were observed in each species. Arabidopsis showed enrichment in protein kinase activity and ion binding, suggesting a focus on signaling and cellular reprogramming. In rice, specific transcripts were associated with mRNA binding and ribosomal components, highlighting an emphasis on translational control, stress response, and energy metabolism. Soybean-specific genes were enriched in transporter and translation activities, with biological processes (BPs) related to growth and symbiotic defense, reflecting its rhizobium-dependent nitrogen fixation and its need to modulate symbiosis under stress. In tomato, enriched functions included phosphoric ester hydrolase activity and processes related to structural and shoot system development, indicating a strategy centered on developmental adaptation (Fig. 4C). Subcellular localization analysis of the proteins encoded by these species-specific transcripts revealed that the majority of encoded proteins were localized to the cytoplasm across all plant species, suggesting that cytoplasmic processes may play a central role in managing MFSC responses (Supplementary Fig. S8B). Overall, while a core set of functional responses was observed, each species exhibited unique transcriptomic signatures shaped by its physiology and evolutionary adaptations to environmental complexity.

**Figure 5.**
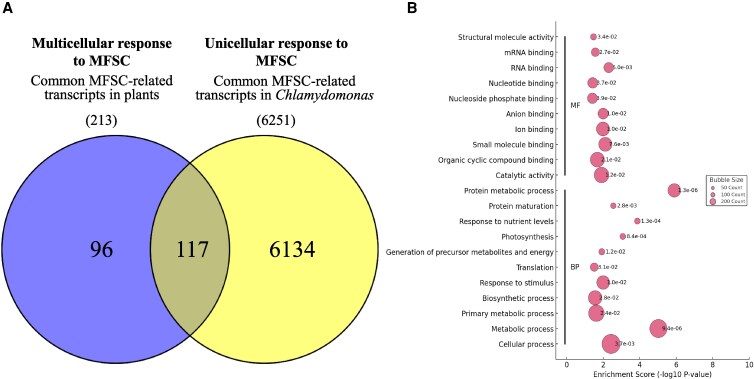
Molecular responses to MFSC in multicellular (tomato, soybean, rice, and Arabidopsis) and unicellular (*C. reinhardtii*) organisms. **A)** Venn diagram showing the overlap between common genes responding to MFSC in multicellular organisms (tomato, soybean, rice, and Arabidopsis; 213, from Fig. 4A) and Arabidopsis homologs of *C. reinhardtii* proteins significantly altered in response to MFSC of 4- and 5-stress combinations (7,432 from Pascual et al. 2025). **B)** GO analysis of the 117 genes common to the response of a unicellular (*C. reinhardtii*) and multicellular (tomato, Arabidopsis, soybean, and rice) photosynthetic organisms to MFSC (from A).

To compare the MFSC response of multicellular plants (Fig. 4) with that of the unicellular alga *Chlamydomonas reinhardtii*, Arabidopsis homologs were first determined for proteins significantly altered in their abundance under 4- and 5-stress conditions in *Chlamydomonas* (Pascual et al. 2025; Supplementary Tables S37 and S38). These were then compared with the list of common MFSC-response genes of Arabidopsis, tomato, rice, and soybean (Fig. 5). Interestingly, a group of 117 transcripts was found to be conserved among *Chlamydomonas* (photosynthetic unicellular), and Arabidopsis, rice, soybean, and tomato (representing photosynthetic multicellular organisms; Fig. 5A, Supplementary Table S39). These genes, enriched under high-complexity MFSC conditions, belonged to functional categories related to translation, nutrient deficiency signaling, primary metabolism, and energy metabolism (Fig. 5B), and were localized to the cytoplasm, as well as to photosynthesis-related structures (Supplementary Fig. S8C), reinforcing the notion of an evolutionary conserved molecular framework activated in response to MFSC.

### Transcriptomic responses to heat stress in tomato plants subjected to MFSC

**Figure 6.**
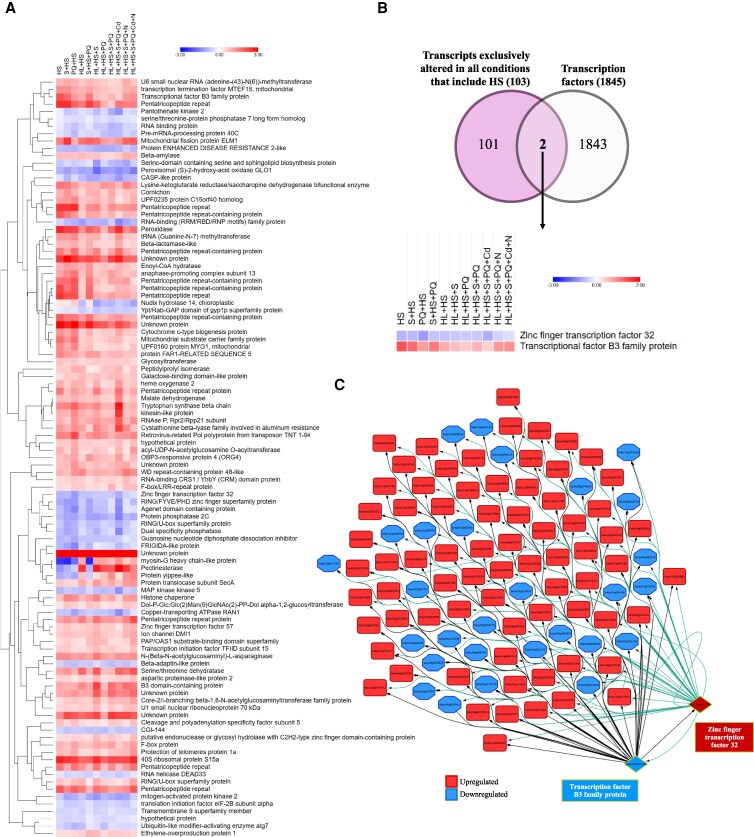
The transcriptomic response to HS during MFSC in tomato plants. **A)** Heat map displaying the expression of 103 transcripts specific to all stress combinations involving HS (but not altered under any other individual or combined stress conditions). **B)** Venn diagram showing the number of TFs specifically altered in response to all stress conditions involving HS (from A; up), and the expression pattern of the 2 TFs exclusively altered under these stress combinations (bottom). **C)** Representative result of the GENIE3 network analysis for the 2 selected TFs shown in B. Upregulated transcripts are depicted in red rectangles, and downregulated transcripts are depicted in blue octagons. Red and blue diamonds represent target TFs (up- and down-regulated, respectively).

Given the significance of HS as a persistent stress common to many regions around the world (due to global warming), and its frequent combination with other stresses (Zandalinas et al. 2021a), we conducted an in-depth analysis of the transcriptomic response of tomato plants subjected to HS and all stress combinations/MFSCs involving HS (Fig. 6). Our analysis identified a total of 103 transcripts that were significantly altered under HS and the different stress combinations/MFSCs that included HS (but not altered in response to any other stress/stress combination/MFSC that did not involve HS; Fig. 6A, Supplementary Table S40). Interestingly, this analysis revealed several transcripts encoding unknown proteins (Solyc04g050180, Solyc06g072075, Solyc07g005250, Solyc09g074500, Solyc10g074750, and Solyc11g012490) that were highly upregulated under all HS-containing conditions, pointing to a potential heat-specific regulatory mechanism involving previously uncharacterized genes that may play a critical role in tomato responses to HS within MFSC scenarios.

To further characterize MFSC-associated HS-responsive transcripts, a Venn diagram was constructed to identify the overlap between HS-specific transcripts and a list of all known tomato TFs (Fig. 6B). Remarkably, this analysis uncovered only two TFs (Zinc finger TF32, Solyc04g017710; and B3 family protein, Solyc08g006230) common among the transcripts responsive to all HS conditions, suggesting a potential central role for these TFs in HS adaptation under MFSC. Furthermore, a network analysis using GENIE3 revealed significant interactions between these two TFs and some of the other 101 HS-specific transcripts (Fig. 6C, Supplementary Table S41). The edges from these two TFs to the other HS-specific transcripts indicate regulatory connections, suggesting a hierarchical gene regulatory structure in which these TFs act as master regulators of HS-related responses in tomato plants (Fig. 6C, Supplementary Table S41).

### Metabolomic responses of tomato plants subjected to MFSC

**Figure 7.**
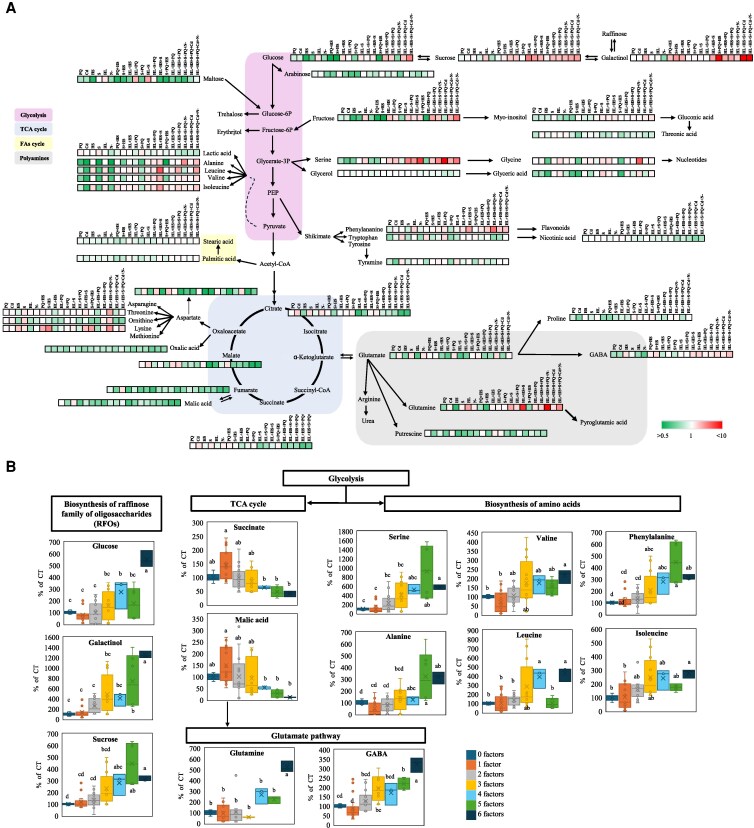
Metabolomic responses of tomato plants subjected to MFSC. **A)** Levels of primary metabolites involved in glycolysis, TCA cycle, fatty acid, and polyamine metabolism in tomato plants subjected to MFSC. **B)** Trends in the biosynthesis of RFOs and amino acids, as well as in the TCA and glutamine metabolic pathways, under increasing complexity of MFSC (1- to 6-stress combinations). Significant metabolite levels (*P* < 0.05) are expressed as fold change compared to control conditions and are shown as a color scale. Detailed information can be found in Supplementary Table S42.

To complement the transcriptomic analysis (Figs. 1 to 6), a targeted metabolomic profiling study was conducted to characterize primary metabolic pathways associated with MFSC in tomato (Fig. 7, Supplementary Fig. S9, and Supplementary Table S42). The accumulation of primary metabolites was assessed across all different stress combinations, revealing significant alterations in central metabolic pathways, including glycolysis, the tricarboxylic acid (TCA) cycle, fatty acid (FA) metabolism, and polyamine biosynthesis. Previous studies reported the accumulation of common metabolites under individual abiotic stresses, including sugars such as sucrose, compatible solutes such as trehalose, polyols, and amino acids including proline, γ-aminobutyric acid (GABA), and branched-chain amino acids (valine, leucine, isoleucine), which are generally elevated as part of the plant protective response and are often described as universal markers of stress due to their roles in osmoprotection, redox balance, and signaling (Obata and Fernie 2012; Bulut et al. 2025). However, in our study, in which individual stresses were applied at low intensity, we observed a contrasting pattern: most stresses, except HS and HL in some cases, led to a decrease in alanine, leucine, valine, and isoleucine. Additionally, sucrose levels showed no major changes, and both GABA and proline generally decreased slightly under individual stresses (Fig. 7, Supplementary Table S42). These findings suggest that the intensity of stress strongly influences metabolic responses, and low-level stresses may not activate the canonical accumulation of protective metabolites response typically reported under higher stress pressure. Interestingly, under 4-, 5-, and 6-stress combinations, a general progressive accumulation of Raffinose Family of Oligosaccharides (RFOs), namely sucrose, glucose, and galactinol, as well as fructose, was observed as the number of stresses increased. This increase could suggest a potential osmoprotective and redox response strategy that mitigates osmotic stress, ROS accumulation, and energy storage deficit under conditions of MFSC. In addition, amino acid profiling revealed increased levels of serine, phenylalanine, isoleucine, lysine, glutamine, and GABA during 4-, 5-, and 6- MFSC. Interestingly, metabolites associated with the TCA cycle (citrate, succinate, malate, and fumarate) were significantly reduced under MFSC conditions (Fig. 7, Supplementary Table S42), pointing to a metabolic trade-off in which plants downregulate energy-intensive pathways to prioritize stress adaptation mechanisms.

### Coordinated transcriptional regulation of stress-responsive metabolic pathways during MFSC

**Figure 8.**
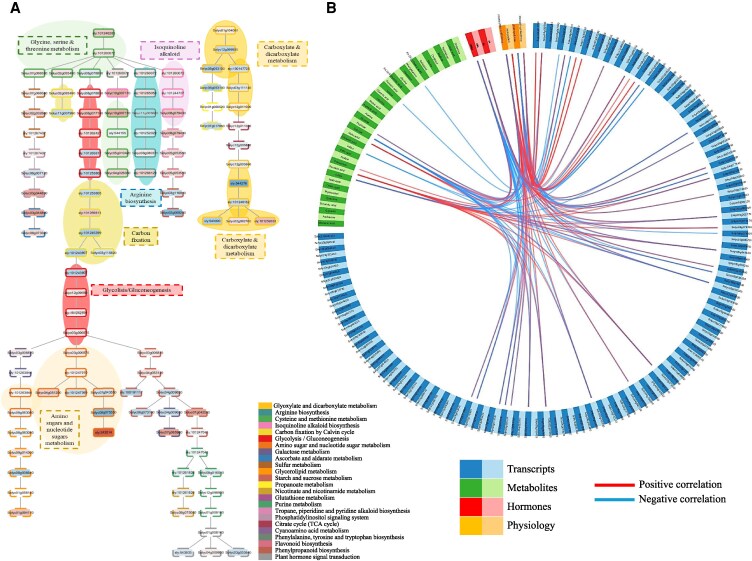
Metabolic and transcriptomic reprogramming in tomato plants subjected to MFSC and mixOmics analysis. **A)** IMPRes analysis of MFSC in tomato. A representative pruned network diagram for the IMPRes in silico pathway analysis of the response of tomato to MFSC. Transcript names are given in nodes, and their expression levels are indicated by the colors red or blue, corresponding to upregulation and downregulation, respectively. Each pathway in the diagram is assigned a different color. The significance of each transcript within a pathway is depicted by the border pattern surrounding the node. Significant transcripts (*P* < 0.05) are depicted with a solid border, whereas non-significant transcripts contain a dashed border. **B)** MixOmics analysis linking transcripts altered under MFSC with metabolomic, hormonal, and physiological changes in tomato plants exposed to MFSC.

Building upon the metabolomic profiles described in Fig. 7, in which key metabolites related to RFO biosynthesis, the TCA cycle, and the glutamate pathway were significantly altered in response to increasing MFSC complexity, we next assessed whether these metabolic shifts were reflected at the transcriptional level. To this end, we performed an Integrative Multi-Omics Pathway Resolution (IMPRes) analysis to identify enriched metabolic pathways based on the transcriptomic dataset (Fig. 8A, Supplementary Table S43). This analysis revealed a significant enrichment in transcripts involved in glycine, serine, and threonine metabolism, as well as arginine biosynthesis, under MFSC, both of which potentially play central roles in nitrogen assimilation and redox balance (Fig. 8A, Supplementary Table S43). The transcriptional responses identified by our IMPRes analysis were consistent with the observed accumulation of amino acids and GABA (Fig. 7), supporting the hypothesis that these pathways contribute to osmoprotection and stress-related nitrogen remobilization under MFSC. In addition, transcripts associated with glycolysis/gluconeogenesis and carbon fixation were also enriched in the response of tomato to MFSC, pointing to a reorganization of central carbon metabolism to accommodate elevated energy demands under stress (Fig. 8A, Supplementary Table S43). In line with these results, the depletion in TCA intermediates observed in Fig. 7 suggests a shift in metabolic fluxes from energy production toward protective biosynthetic processes. In addition, enrichment in amino sugar and nucleotide sugar metabolism likely reflects cell wall remodeling, while the overrepresentation of carboxylate and dicarboxylate metabolism supports the involvement of TCA-derived organic acids in stress adjustment. Finally, enrichment in the isoquinoline alkaloid biosynthesis pathway suggests possible activation of specialized secondary metabolism, potentially linked to detoxification and cellular defense. Taken together, the results shown in Fig. 8A and Supplementary Table S43 demonstrate that MFSC triggers coordinated transcriptional changes in both primary and secondary metabolic networks, reinforcing the link between gene regulation and metabolic adaptation under complex stress conditions of MFSC.

### Multi-omics integration reveals key molecular, metabolic, and physiological interactions during tomato stress adaptation to MFSC

**Figure 9.**
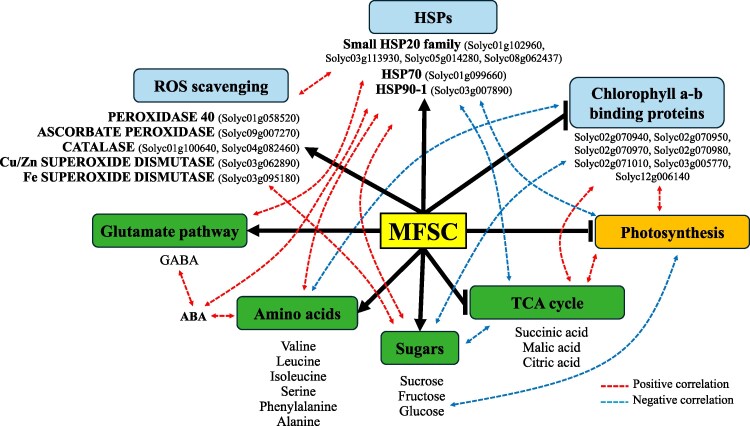
A schematic model of tomato adaptation strategies to conditions of MFSC, highlighting key interactions between transcriptional regulators, physiological changes, metabolic pathways, and hormonal signaling in response to MFSC. MFSC is depicted as a yellow rectangle at the center of the network. Blue and green rectangles indicate transcripts and metabolic pathways or metabolites identified in the present study, respectively. The orange rectangle represents the photosynthetic rate (based on Pascual et al. 2023) as a physiological parameter. Solid black arrows and inhibitory T-bars represent direct regulatory interactions whereby MFSC enhances or represses, respectively, the connected transcripts, metabolic pathways, or physiological traits. Dashed arrows indicate correlations predicted by our integrated multi-omics correlation analyses (using IMPRes and mixOmics). The dashed arrows are colored by the sign of the correlation: red dashed arrows denote positive correlations, and blue dashed arrows denote negative correlations (as detailed in Supplementary Table S44).

To establish functional linkages between transcriptomic, metabolic, and physiological responses of tomato plants subjected to MFSC, a mixOmics analysis was performed (Fig. 8B, Supplementary Table S44). This integrative approach allowed the identification of correlated gene–metabolite–physiology interactions that underline adaptive strategies of tomato plants to MFSC. As inputs, significantly altered transcripts and primary metabolites in response to MFSC (6-stress combination), physiological data (including photosynthetic rate, stomatal conductance, transpiration rate, and PS_II_ efficiency; from Pascual et al. 2023), and levels of different hormones (jasmonic acid [JA], salicylic acid [SA], ABA, and oxylipin-12-oxo-phytodienoic acid [OPDA]; from Pascual et al. 2023), were used. This analysis revealed that one of the main hormones involved in abiotic stress signaling, i.e. ABA, displayed a tight correlation with the expression pattern of different transcripts including members of the small HSP20 family (Solyc01g102960, Solyc03g113930, Solyc05g014280, Solyc08g062437), HSP70 (Solyc01g099660), or HSP90-1 (Solyc03g007890), as well as with the accumulation of GABA, phenylalanine, isoleucine, and alanine metabolites. In addition, the expression of different Chlorophyll a-b binding proteins (CAB proteins; Solyc02g070940, Solyc02g070950, Solyc02g070970, Solyc02g070980, Solyc02g071010, Solyc03g005770, Solyc12g006140) was positively correlated with photosynthetic rate, PS_II_ efficiency, and the accumulation of different intermediates of the TCA cycle, but negatively correlated with the accumulation of sugars and amino acids (Figs. 8B and 9, Supplementary Table S44), indicating a potential important role of these proteins in maintaining or enhancing the photosynthetic process under MFSC conditions. In addition, different transcripts encoding ROS scavenging enzymes (e.g. Peroxidase 40 [Solyc01g058520], ascorbate peroxidase [Solyc09g007270], catalase [Solyc01g100640, solyc04g082460], Cu/Zn superoxide dismutase [Solyc03g062890], and Fe superoxide dismutase [Solyc03g095180]) that accumulated under MFSC, were positively correlated with the accumulation of HSPs (Figs. 8B and 9, Supplementary Table S44).

## Discussion

Understanding how plants respond to MFSC is increasingly critical, as climate change and enhanced pollution levels increase the simultaneous occurrence of multiple abiotic stressors in our environment (Rillig et al. 2019; Zandalinas et al. 2021a; IPCC 2022; Zandalinas and Mittler 2022). While extensive research explored the effects of individual and 2-stress combinations on plants, plant responses to higher-order stress complexity remained poorly understood. Unlike isolated stress conditions, MFSC disrupts plant homeostasis through concurrent perturbations of multiple signaling pathways, metabolism, and hormonal networks, requiring a more coordinated and adaptive response. Previous MFSC studies in *A. thaliana*, *O. sativa*, *G. max*, and *C. reinhardtii* identified both conserved and species-specific molecular signatures (Zandalinas et al. 2021b; Peláez-Vico et al. 2024; Sinha et al. 2024; Pascual et al. 2025). Here, we present a comprehensive transcriptomic, metabolomic, and physiological analysis of tomato (*S. lycopersicum*) exposed to MFSC involving up to 6 stressors (high light, heat, salinity, paraquat, Cd, and N deficiency). This study represents a metabolomic analysis of plant responses to MFSC and provides insights into how plants orchestrate stress-specific adaptations under increasingly complex environmental stress conditions.

Our transcriptomic findings indicate a higher degree of molecular reprogramming as stress complexity increased, with over 6,000 transcripts commonly altered under 4-, 5-, and 6-MFSC conditions, consistent with prior observations in Arabidopsis and soybean (Zandalinas et al. 2021b; Peláez-Vico et al. 2024). GO enrichment analysis of this set of transcripts highlighted alterations in key metabolic pathways such as glycolysis, carbon fixation, and dicarboxylate metabolism, suggesting metabolic shifts in cellular resource allocation to maintain homeostasis under complex stress conditions (Fig. 2A). This response contrasts with that observed in soybean leaves responding to MFSC, where transcriptomic enrichment was primarily associated with ribosome function and structure, translation, and RNA processing (Peláez-Vico et al. 2024). Interestingly, despite the heterogeneity among the different stresses/stress combination responses, a conserved set of 194 transcripts (12 of which were TFs) was commonly altered across all stress conditions (Figs. 2, B, and 3, A, B), revealing the potential existence of a core MFSC molecular signature that could emerge as an important trait of high biotechnological relevance. In addition, we identified a specific reprogramming in the expression of 155 TFs under MFSC that highlighted the regulatory complexity associated with MFSC responses. Notably, combinations involving four or more stressors showed strong enrichment in key TF families such as WRKY, NAC, MYB, and ERF (Fig. 3, C and D). While some of these TFs, including DREB1 and DREB2 (Liu et al. 1998; Kidokoro et al. 2015; Hichri et al. 2016), are known as general stress regulators, the exclusive upregulation of 155 TFs under the most complex MFSC conditions supports the hypothesis that plant responses to stress enhance not only in magnitude but also in regulatory specificity as environmental challenge increases. This complexity-dependent alteration in TF expression likely allows the fine-tuned integration of hormonal and environmental signals, further supported by the concurrent modulation of hormone (Pascual et al. 2023)- and ROS (Supplementary Figs. S4 and S5)-related pathways. The evolutionary dimension of our analysis reinforces the functional relevance of the identified MFSC-related transcripts. We identified 213 MFSC-responsive transcripts conserved among tomato, Arabidopsis, soybean, and rice (Fig. 4A), with 117 also shared with the unicellular green alga *C. reinhardtii* (Fig. 5A). Functional annotation of these conserved transcripts points to roles in translation, nutrient signaling, primary metabolism, and energy metabolism, suggesting that responses to complex stress conditions could have ancient evolutionary roots (Fig. 5B). Such cross-species conservation further suggests that key stress-response elements of the MFSC response have been retained since before the divergence of land plants (Merchant et al. 2007), highlighting their potential as universal biomarkers and promising targets for bioengineering of climate-resilient crops.

Heat stress has been recognized as a dominant factor in stress combinations due to its adverse effects on protein folding, membrane integrity, and enzymatic activity (Kotak et al. 2007; Mittler et al. 2012; Balfagón et al. 2019; Kan et al. 2023), and in our data, its inclusion led to more pronounced transcriptomic changes than other factors (e.g. the altered expression of HSPs; Supplementary Fig. S3). Notably, we identified 103 transcripts uniquely regulated under MFSC scenarios involving HS, five of which are uncharacterized, potentially representing heat-specific stress-responsive markers (Fig. 6A). Our analysis further revealed two transcription factors (the Zinc finger TF 32 and a B3-domain TF), as key regulators of these heat-specific responses (Fig. 6, B and C). Their influence on HS-specific transcriptional modules and previous associations with stress tolerance in legumes and soybean (Kiełbowicz-Matuk 2012; Ren et al. 2023; Huang et al. 2023) further support their relevance as candidate genes for thermotolerance breeding in tomato. To determine the molecular role of these TFs in HS tolerance, future experiments should include gene overexpression and CRISPR-Cas9-mediated knockout studies in tomato to assess their functional impact on thermotolerance. Transcriptomic and hormonal profiling of such modified lines under HS-involving MFSC conditions would help clarify their downstream regulatory networks and integration with key signaling pathways. Additionally, field-based phenotyping of modified lines under natural heat episodes would be essential to validate their utility under realistic climate scenarios. Such studies will be crucial to translate molecular insights into applied/commercial strategies for breeding heat-resilient tomato varieties and, potentially, other crops facing increasing climate challenges.

Our metabolic analysis revealed marked accumulation of osmoprotectants such as galactinol, glucose, fructose, and sucrose under MFSC (Fig. 7), in line with their known role in osmoprotection, stabilizing proteins and membranes, and buffering cellular redox status (Nishizawa et al. 2008). Interestingly, we also observed a significant and progressive accumulation of specific amino acids (particularly glutamine, serine, phenylalanine, lysine, and GABA) as the number of stress factors increased (Fig. 7). This trend was further supported by IMPRes analysis, which identified the activation of amino acid metabolism under MFSC (Fig. 8A), suggesting enhanced nitrogen remobilization and activation of the GABA shunt in response to MFSC. This metabolic change could indicate an alteration in carbon-nitrogen balance, typical under energy-deficient conditions resulting from suppressed photosynthesis under MFSC (Pascual et al. 2023), and may contribute to ROS scavenging (Supplementary Fig. S5) and metabolic flexibility (Bouché and Fromm 2004; Fait et al. 2008). In contrast, the levels of TCA cycle intermediates, such as malate, succinate, and citrate progressively declined with the increasing stress complexity (Fig. 7). These reductions showed strong positive correlation with the expression of photosynthesis-related transcripts such as CAB proteins (Figs. 8B and 9), as well as with photosynthetic rates under MFSC conditions (Pascual et al. 2023). Our IMPRes pathway enrichment analysis further revealed a significant overrepresentation of transcripts associated with carboxylate and dicarboxylate metabolism in the MFSC transcriptomic dataset (Fig. 8A). Taken together, these findings suggest that the increase in stress severity/complexity under MFSC leads to a metabolic shift that limits carbon flow through the TCA cycle, likely as a result of impaired photosynthetic activity and reduced carbon assimilation (Fig. 9). The coordinated downregulation of TCA intermediates and photosynthetic transcripts, together with the alteration of carboxylate metabolism pathways, may reflect a reprogramming of central metabolism toward stress acclimation rather than biomass accumulation. Consistently, the observed suppression of photosynthesis was paralleled by reduced plant growth and significant morphological damage under MFSC (Supplementary Fig. S10). This could involve the redirection of carbon skeletons for alternative uses (e.g. amino acid biosynthesis), or a strategy to conserve energy and resources under complex environmental stress combinations. Moreover, the increased leaf damage observed under conditions of MFSC (Supplementary Fig. S10) coincided with higher oxidative stress, as evidenced by elevated malondialdehyde (a marker of lipid peroxidation) accumulation observed in previous works (Pascual et al. 2023), and the strong upregulation of genes encoding antioxidant enzymes such as peroxidases, catalases, and superoxide dismutases (Supplementary Fig. S5), as well as HSPs (HSP20s, HSP70, HSP90; Supplementary Fig. S3), and the hormone ABA (Pascual et al. 2023). These findings strongly suggest that under complex stress conditions, tomato plants activate tightly coordinated detoxification and defense mechanisms aimed at mitigating cellular damage and preserving metabolic homeostasis. However, while these responses are essential for survival, their high energetic cost and associated metabolic reprogramming likely contribute to the observed reduction in photosynthetic efficiency, TCA cycle activity, and overall growth, revealing potential trade-offs between stress tolerance and plant performance under MFSC. Previous reports of MFSC studies in Arabidopsis, soybean, and rice (Zandalinas et al. 2021b; Peláez-Vico et al. 2024; Sinha et al. 2024), revealed that ROS detoxification and redox regulation were shown to be key components of the acclimation response to these conditions. These results support the notion that ROS detoxification is a conserved and critical mechanism across different species in enhancing plant resilience to complex environmental stress. Future research on tomato could explore the functional characterization of key antioxidant genes or employ genetic approaches to manipulate redox-regulatory pathways as potential strategies to improve tolerance to MFSC.

Altogether, our multi-omics study suggests that the response of tomato plants to MFSC is not simply an additive effect of individual stress pathways, but rather a qualitatively distinct process involving unique transcriptional, metabolic, and physiological signatures that are coordinated, partially conserved, and dependent on stress complexity. The emergence of specialized TFs, the accumulation of protective metabolites, and the suppression of growth-related processes suggest that plants possess an inherent ability to detect environmental complexity and alter their internal networks accordingly. These findings have significant implications for agriculture under climate change, as they suggest that improving crop resilience will require engineering or selecting for traits that enable robust coordination of hormonal signaling, stress perception, and metabolic reprogramming under complex environmental scenarios. Finally, the identification of core conserved transcripts and metabolites that define the MFSC response provides a valuable resource for future breeding programs aimed at enhancing plant performance under unpredictable and MFSC. However, further studies are needed to functionally validate the core conserved transcripts and metabolites related to MFSC response. Targeted gene editing, as well as studies of transcriptomics, metabolic flux analysis, and integrative omics approaches in edited lines, could reveal their mechanistic roles in coordinating plant adaptation to complex stress environments. These experiments will be a key to translating core molecular signatures into actionable strategies for improving crop resilience.

## Materials and methods

### Plant material, stress treatments, and experimental design

Tomato plants were grown and subjected to MFSC as previously described by Pascual et al. (2023) and shown in Fig. 1. Briefly, wild-type Moneymaker seeds were sown in a substrate composed of 80% peat moss, 10% perlite, and 10% vermiculite, under greenhouse conditions with 70% relative humidity, natural photoperiod, 200 *µ*mol photons m^−2^s^−1^ light intensity, and day and night temperature averaging 25.0 ± 3.0 °C and 18.0 ± 3.0 °C, respectively. Temperature and relative humidity were recorded regularly with a portable USB datalogger (OM-EL-WI- N-USB, Omega, NJ, USA). Once germinated, seedlings grew under greenhouse conditions and were watered with half-strength Hoagland solution for 7 d. After that, stress combinations of up to 6 factors including high light (700 *μ*mol m^−2^ s^−1^; HL), heat stress (37 °C; HS), salinity (75 mm NaCl; S), nitrogen deficiency (Ca(NO₃)₂ concentration was reduced by 90%; N^−^), heavy metal stress (10 *µ*M CdSO_4_; Cd), and the herbicide paraquat (1 *µ*M PQ) were imposed on 8 plants per stress treatment (Fig. 1), and MFSC experiments were repeated 3 times. As shown in Fig. 1, a group of tomato plants was subjected to N^−^ by irrigating with half-strength Hoagland solution containing 10% of the N concentration, supplied as Ca(NO₃)₂. After 1 wk of N^−^ treatment, separate groups of plants were exposed to the following stress conditions for a duration of 15 d: PQ (1 *µ*M PQ), S (75 mm NaCl), Cd (10 *µ*M CdSO_4_), S + PQ (75 mm NaCl + 1 *µ*M PQ), S + PQ + N^−^ (75 mm NaCl + 1 *µ*M PQ + 10% N), S + PQ + Cd (75 mm NaCl + 1 *µ*M PQ + 10 *µ*M CdSO_4_), and S + PQ + Cd + N^−^ (75 mm NaCl + 1 *µ*M PQ + 10 *µ*M CdSO_4_ + 10% N). For stress combinations involving HS and/or HL, plants were exposed to a 9-hour treatment in growth chambers at 37 °C (HS) and/or 700 *μ*mol m⁻² s⁻¹ light intensity (HL). Therefore, HS, HL, S, and PQ stresses were conducted in all possible combinations, and N^−^ and Cd were added as single stresses, as well as in combination with HL +HS + S + PQ to generate 2 different 5-stress and 1 6-stress combinations, as described by Pascual et al. (2023) and similar to Rillig et al. (2019); Zandalinas et al. (2021b); Peláez-Vico et al. (2024), and Sinha et al. (2024). Young leaves were sampled at the end of all stresses (Fig. 1) and were stored at −80 °C until further analysis.

### Metabolic profiling

Levels of polar metabolites were determined as described by Vazquez-Vilar et al. (2023). Approximately 85 mg of fresh plant tissue was weighed and extracted with 1.4 mL of absolute methanol, supplemented with 60 *µ*L of an aqueous ribitol solution (0.2 mg mL⁻¹) used as an internal standard. Metabolite extractions were performed at 70 °C for 15 min in a water bath. The extract was centrifuged (14,000 rpm, 10 min), and the supernatant was recovered and fractionated by adding chloroform and Milli-Q water. After vigorous vortex and centrifugation at 4,000 rpm, 150 *µ*L of the aqueous phase were recovered and dried overnight under vacuum in a centrifuge concentrator (Speed Vac, Jouan, Saint Herblain Cedex, France). The dry residue was subjected to a double derivatization procedure with methoxyamine hydrochloride (20 mg mL^−1^ in pyridine, Sigma) and N-Methyl-N-(trimethylsilyl)trifluoroacetamide (Macherey-Nagel). Fatty acid methyl esters (C8-C24) were added and used as retention index markers. Analyses were performed on a 6890N gas chromatograph (Agilent Technologies, USA) coupled to a Pegasus 4D TOF mass spectrometer (LECO, St. Joseph, MI). Chromatography was performed with a BPX35 (30 m, 0.32 mm, 0.25 *µ*m) capillary column (SGE Analytical Science Pty Ltd., Australia) with a 2 mL min^−1^ helium flow. Oven programming conditions were established as follows: 2 min of isothermal heating at 85 °C, followed by a 15 °C min^−1^ temperature ramp up to 360 °C. The injection temperature was set at 230 °C, and the ion source was adjusted to 250 °C. Data were acquired after electron ionization at 70 eV, and recorded in the 70–600 m/z range at 20 scans s^−1^. Chromatograms were analyzed by means of ChromaTOF software. Metabolites were identified by comparison of both mass spectra and retention time with those of pure standards injected under the same conditions. The peak area of each identified compound was normalized to the internal standard area and the exact sample weight, followed by normalization relative to the control treatment. Three independent biological repeats per stress group were performed.

### Transcriptomics

To perform the transcriptomic analysis of MFSC on tomato plants, RNA was extracted from all the different stress conditions using an RNeasy Mini kit (Qiagen, Hilden, Germany) following the manufacturer's instructions. Total RNA concentration and purity were determined using a Nanodrop 2000 spectrophotometer (Thermo Scientific, Wilmington, DE, USA) from the ratio of absorbance readings at 260 and 280 nm. RNA sequencing was performed by Novogene Sequencing Europe facilities (London, UK). Briefly, single-end sequenced reads were quality tested using FASTQC v.0.11.7 (Andrews 2010) and aligned to the reference genome of Tomato (genome build 10) obtained from Solgenomics (https://solgenomics.net/) using STAR ALIGNER v.2.4.0.1 (Dobin et al. 2013). Default mapping parameters (10 mismatches/read; 9 multi-mapping locations/read) were used. The genome index was generated using the gene annotation file obtained from Solgenomics. Differential gene expression analysis was carried out using DESEQ2, an R-based package available from BIOCONDUCTOR (Love et al. 2014), with mapped read counts generated using STAR ALIGNER v.2.4.0.1 (Dobin et al. 2013). Genes differentially expressed were identified by comparing the mapped read abundance under the different conditions against CT. Gene expression was measured as mean normalized counts of reads mapped onto the different genes (Love et al. 2014). The difference in expression was quantified in terms of the logarithm (log_2_) of the ratio of mean normalized counts between two conditions (log fold change). Differentially expressed genes were defined as those with an adjusted *P*-value < 0.05 (negative binomial Wald test followed by the Benjamini–Hochberg correction; Love et al. 2014). Genes with 0 raw fold-change expression value were omitted from further analysis. Differentially expressed genes were classified into upregulated or downregulated based on significant positive or negative log fold-change values, respectively. Functional annotation and quantification of overrepresented GO terms (*P* < 0.05) were conducted using DAVID Bioinformatics Resources 6.8 (https://davidbioinformatics.nih.gov/; Huang et al. 2009). Venn diagrams were created in VENNY 2.1 (BioinfoGP, CNB-CSIC), UpSet plots were generated in UpsetR (gehlenborglab.shinyapps.io; Conway et al. 2017), and heatmaps were represented by Morpheus software (https://software.broadinstitute.org/morpheus). To identify targets of selected transcription factors, the R package GENIE3 1.18.0 (Huynh-Thu et al. 2010) was used, employing the Random Forest tree calculation method and a weight cutoff of 0.42, as described by Peláez-Vico et al. (2024).

### Promoter analysis for transcription factor binding sites

A total of 2,698 transcripts specifically regulated under the 4-, 5-, and 6-factor stress conditions were selected for cis-regulatory motif analysis. Promoter sequences were retrieved from the *S. lycopersicum* reference genome available at SolGenomics. For each gene, the promoter was defined as the region spanning from 1,500 base pairs upstream to 100 base pairs downstream of the annotated transcription start site. In cases where genes were located near chromosomal termini, the promoter sequence was truncated to the maximum available upstream region. Strand orientation was considered to ensure accurate promoter extraction for both forward and reverse strand genes. The analysis focused on 5 TF families: WRKY, NAC, ERF (AP2/ERF domain), MYB, and HSF. For each TF family, representative position weight matrices (PWMs) were obtained from the PlantTFDB and JASPAR Plant databases. All PWMs were converted to MEME Suite-compatible formats prior to scanning. Motif discovery was performed using Find Individual Motif Occurrences, part of the MEME Suite (v5.x). Both DNA strands were scanned to identify motif instances corresponding to the selected PWMs. A significance threshold of *P* < 10^−4^ was applied to retain only high-confidence motif occurrences, thereby minimizing the inclusion of potential false positives.

### IMPRes in silico hypothesis generation

To identify active pathways from complex multi-omics data, the algorithm IMPRes (Jiang et al. 2020a,b) was used. For this analysis, transcriptomic data for control and the different stress combinations, along with 10 input seed genes identified as significant from differential expression analysis, were integrated with the tomato pathway background network constructed from KEGG KGML files. The resulting pruned network diagram highlights the interconnections among active pathways, illustrating how the seed genes are functionally linked to other genes within these pathways.

### Multi-omics integrative correlation analyses

The mixOmics (v3.21; Rohart et al. 2017) R package was employed to associate transcriptomic data with metabolomic, hormonal, and physiological changes of tomato plants exposed to MFSC. The transcriptomic data were the combination of differentially expressed genes identified with a *q*-value less than 0.05 and log_2_ fold change (log_2_FC) of ≥ 7 for all comparisons. This strict criterion captured genes with strong differential expressions under the different stress combination treatments. Only genes that passed these criteria in at least one condition were retained, followed by removing duplicates to produce the final list of transcripts used for integrative analysis. The physiological data used included photosynthetic rate, stomatal conductance, transpiration rate, and PS_II_ efficiency from Pascual et al. (2023). The hormones used were JA, SA, ABA, and OPDA from Pascual et al. (2023). The metabolites used for this analysis can be found in Supplementary Table S42. Data integration and classiﬁcation were accomplished by Data Integration Analysis and Biomarker discovery using Latent variable approaches for Omics studies (DIABLO). The N-integration Sparse Partial Least Square Discriminant Analysis with function block.splsda was used to identify signatures composed of highly correlated variables across the different matrix sets, allowing the detection of a conﬁdent relationship between the data sets. The circos correlation matrix generated by the circosplot function was imported to the circlize (Gu et al. 2014) R package to create the circosplot. The gene expression correlated with metabolomic, physiological, and hormonomic datasets with a correlation cutoff of 0.7.

### Statistical analysis

Results are presented as the mean ± SE. Statistical analysis was performed with Statgraphics Plus v.5.1. software (Statistical Graphics Corp., Herndon, VA, USA) by 2-way ANOVA followed by Tukey post hoc test (different letters denote statistical significance at *P*  *<* 0.05). For results shown as Box and Whisker plots, borders correspond to the 25th and 75th percentiles of the data, and the center line indicates the median.

### Accession numbers

Sequence data from this article can be found in the Sol Genomics data libraries under accession numbers Solyc01g102960, Solyc03g113930, Solyc05g014280, Solyc08g062437, Solyc01g099660, Solyc03g007890, Solyc02g080410, Solyc02g080470, Solyc03g113180, Solyc01g103920, Solyc01g088200, Solyc11g071610, Solyc01g058520, Solyc09g007270, Solyc01g100640, Solyc04g082460, Solyc03g062890, Solyc03g095180, Solyc01g066980, Solyc09g083290, Solyc03g123600, Solyc02g082633, Solyc02g081170, Solyc01g104900, Solyc05g007420, Solyc06g060830, Solyc07g006630, Solyc01g109880, Solyc07g052700, Solyc04g017710, and Solyc08g006230.

## Supplementary Material

kiaf519_Supplementary_Data

## Data Availability

RNA-Seq data were deposited in Gene Expression Omnibus (GEO), under the following accession number: GSE294808.

## Supplementary data

The following materials are available in the online version of this article.

**Supplementary Figure S1.** PCA showing differences in transcriptomic responses of *S. lycopersicum* to MFSC.

**Supplementary Figure S2.** Subcellular localization of MFSC-responsive transcripts.

**Supplementary Figure S3.** Heat map displaying the expression patterns of HSPs across all stress conditions.

**Supplementary Figure S4.** Heat map displaying the expression patterns of transcripts related to ROS production across all stress conditions.

**Supplementary Figure S5.** Heat map displaying the expression patterns of transcripts related to ROS scavenging across all stress conditions.

**Supplementary Figure S6.** Complete heat map displaying the expression patterns of commonly altered transcripts across all stress conditions.

**Supplementary Figure S7.** Complete heat map displaying the expression patterns of transcription factors specifically altered in response to 4-, 5-, or 6-stress combinations and not altered under other conditions.

**Supplementary Figure S8.** Subcellular localization of MFSC-responsive transcripts in multicellular (tomato, soybean, rice, and Arabidopsis) and unicellular (*C. reinhardtii*) organisms. A) A GO enrichment analysis of the cellular component category of the 213 genes commonly altered in tomato, Arabidopsis, soybean, and rice in response to MFSC (from Fig. 4, A and B). B) A GO enrichment analysis of the cellular component category of transcripts specifically altered in each species under MFSC (2,307 in Arabidopsis, 162 in rice, 6,300 in soybean, and 727 in tomato; from Fig. 4, A and C). C) A GO enrichment analysis of the cellular component category of the 117 genes common to the response of a unicellular (*C. reinhardtii*) and multicellular (tomato, Arabidopsis, soybean, and rice) photosynthetic organisms to MFSC (from Fig. 5).

**Supplementary Figure S9.** PCA showing differences in metabolomic responses of *S. lycopersicum* to MFSC.

**Supplementary Figure S10.** Growth and morphological parameters in tomato plants exposed to MFSC.

**Supplementary Table S1.** Genes significantly altered compared to control (*P* < 0.05) in *S. lycopersicum* plants in response to PQ.

**Supplementary Table S2.** Genes significantly altered compared to control (*P* < 0.05) in *S. lycopersicum* plants in response to S.

**Supplementary Table S3.** Genes significantly altered compared to control (*P* < 0.05) in *S. lycopersicum* plants in response to HS.

**Supplementary Table S4.** Genes significantly altered compared to control (*P* < 0.05) in *S. lycopersicum* plants in response to N^−^.

**Supplementary Table S5.** Genes significantly altered compared to control (*P* < 0.05) in *S. lycopersicum* plants in response to Cd.

**Supplementary Table S6.** Genes significantly altered compared to control (*P* < 0.05) in *S. lycopersicum* plants in response to HL.

**Supplementary Table S7.** Genes significantly altered compared to control (*P* < 0.05) in *S. lycopersicum* plants in response to S + PQ.

**Supplementary Table S8.** Genes significantly altered compared to control (*P* < 0.05) in *S. lycopersicum* plants in response to S + HS.

**Supplementary Table S9.** Genes significantly altered compared to control (*P* < 0.05) in *S. lycopersicum* plants in response to PQ + HS.

**Supplementary Table S10.** Genes significantly altered compared to control (*P* < 0.05) in *S. lycopersicum* plants in response to HL + S.

**Supplementary Table S11.** Genes significantly altered compared to control (*P* < 0.05) in *S. lycopersicum* plants in response to HL + PQ.

**Supplementary Table S12.** Genes significantly altered compared to control (*P* < 0.05) in *S. lycopersicum* plants in response to HL + HS.

**Supplementary Table S13.** Genes significantly altered compared to control (*P* < 0.05) in *S. lycopersicum* plants in response to S + PQ + HS.

**Supplementary Table S14.** Genes significantly altered compared to control (*P* < 0.05) in *S. lycopersicum* plants in response to HL + S + PQ.

**Supplementary Table S15.** Genes significantly altered compared to control (*P* < 0.05) in *S. lycopersicum* plants in response to HL + HS + S.

**Supplementary Table S16.** Genes significantly altered compared to control (*P* < 0.05) in S*. lycopersicum* plants in response to HL + HS + PQ.

**Supplementary Table S17.** Genes significantly altered compared to control (*P* < 0.05) in *S. lycopersicum* plants in response to HL + HS + S + PQ.

**Supplementary Table S18.** Genes significantly altered compared to control (*P* < 0.05) in *S. lycopersicum* plants in response to HL + HS + S + PQ + Cd.

**Supplementary Table S19.** Genes significantly altered compared to control (*P* < 0.05) in *S. lycopersicum* plants in response to HL + HS + S + PQ + N^−^.

**Supplementary Table S20.** Genes significantly altered compared to control (*P* < 0.05) in *S. lycopersicum* plants in response to HL + HS + S + PQ + N^−^+Cd.

**Supplementary Table S21.** List of common transcripts altered in response to all individual stress conditions in *S. lycopersicum* (Fig. 2A).

**Supplementary Table S22.** List of common transcripts altered in response to all 2-factor stress conditions in *S. lycopersicum* (Fig. 2A).

**Supplementary Table S23.** List of common transcripts altered in response to all 3-factor stress conditions in *S. lycopersicum* (Fig. 2A).

**Supplementary Table S24.** List of common transcripts altered in response to 4-, 5-, or 6-factor stress conditions in *S. lycopersicum* (Fig. 2A).

**Supplementary Table S25.** List of common transcripts altered in response to all stress conditions in *S. lycopersicum* (Fig. 2B).

**Supplementary Table S26.** List of common transcription factors altered in response to all stress conditions in *S. lycopersicum* (Fig. 3A).

**Supplementary Table S27.** List of 2,698 transcripts commonly altered in response to 4-, 5-, or 6-stress conditions and not expressed under any other stress conditions in *S. lycopersicum* (Fig. 3C).

**Supplementary Table S28**. List of transcription factors specifically altered in response to 4-, 5-, or 6-stress conditions in *S. lycopersicum* (Fig. 3C).

**Supplementary Table S29**. List of transcripts commonly altered in response to 4-, 5-, or 6-stress conditions and not expressed under any other stress conditions in *S. lycopersicum* that contain WRKY, NAC, MYB, HSF, and/or ERF transcription factor binding sites in their promoter regions (from Supplementary Table S27).

**Supplementary Table S30**. *A. thaliana* homologs of tomato transcripts significantly altered in response to 4-, 5-, or 6- stress combinations (from Supplementary Table S24) (Fig. 4A).

**Supplementary Table S31**. List of transcripts significantly altered in response to 4-, 5-, or 6-stress combinations in *A. thaliana* (data extracted from Peláez-Vico et al. 2024 and Zandalinas et al. 2021b) (Fig. 4A).

**Supplementary Table S32**. List of proteins significantly altered in response to 4- or 5-stress combinations in rice (data extracted from Sinha et al. 2024) (Fig. 4A).

**Supplementary Table S33**. *A. thaliana* homologs of rice proteins significantly altered in response to 4- or 5-stress combinations (from Supplementary Table S32) (Fig. 4A).

**Supplementary Table S34**. List of transcripts significantly altered in response to 4- or 5-stress combinations in soybean (data extracted from Peláez-Vico et al. 2024) (Fig. 4A).

**Supplementary Table S35**. *A. thaliana* homologs of soybean transcripts significantly altered in response to 4- or 5-stress combinations (from Supplementary Table S34) (Fig. 4A).

**Supplementary Table S36**. List of 213 genes commonly altered among tomato, Arabidopsis, rice, and soybean plants in response to MFSC (Fig. 4A).

**Supplementary Table S37**. List of proteins significantly altered in response to 4- or 5-stress combinations in wild-type *C. reinhardtii* (data extracted from Pascual et al. 2025) (Fig. 5A).

**Supplementary Table S38**. *A. thaliana* homologs of *C. reinhardtii* proteins significantly altered in response to 4- or 5-stress combinations (from Supplementary Table S37) (Fig. 5A).

**Supplementary Table S39**. List of 117 common genes significantly altered in response to MFSC between wild-type *C. reinhardtii* (from **Supplementary Table S38**) and multicellular plants (tomato, Arabidopsis, rice, and soybean; from Supplementary Table S36) (Fig. 5A).

**Supplementary Table S40**. List of transcripts specific to all HS-containing conditions and not expressed under any other stress conditions in *S. lycopersicum* (Fig. 6A).

**Supplementary Table S41**. GENIE3-inferred regulatory links between 2 HS-specific TFs (Zinc Finger TF32, Solyc04g017710; and a B3-domain protein, Solyc08g006230) and 101 HS-specific target transcripts under MFSC in *S. lycopersicum* (Fig. 6C).

**Supplementary Table S42**. Normalized expression of metabolites under MFSC in *S. lycopersicum* (Fig. 7).

**Supplementary Table S43**. IMPRes in silico pathway analysis of the response of tomato plants to 6-factor MFSC (Fig. 8A).

**Supplementary Table S44**. MixOmics analysis linking selected physiological and phytohormonal data (from Pascual et al. 2023), specific transcriptomic data (Supplementary Tables S1 to S20), and metabolomic analysis (Supplementary Table S42) in *S. lycopersicum* plants in response to 6-factor MFSC (Fig. 8B).

## Dive Curated Terms

The following phenotypic, genotypic, and functional terms are of significance to the work described in this paper:

NAC CHEBI: CHEBI:7421

JASPAR Gramene: https://jaspar.elixir.no/
JASPAR Araport: https://jaspar.elixir.no/
