# Supplementary material for: Multi-omics-based insights into tomato adaptation to multifactorial stress combination
Source: Plant Physiol. 2025 Oct 14;199(3):kiaf519. doi: 10.1093/plphys/kiaf519 (PMC12579106; doi:10.1093/plphys/kiaf519)
Supplement: kiaf519_Supplementary_Data [file kiaf519_supplementary_data.zip › Supplementary Figures_EDITED.pdf]

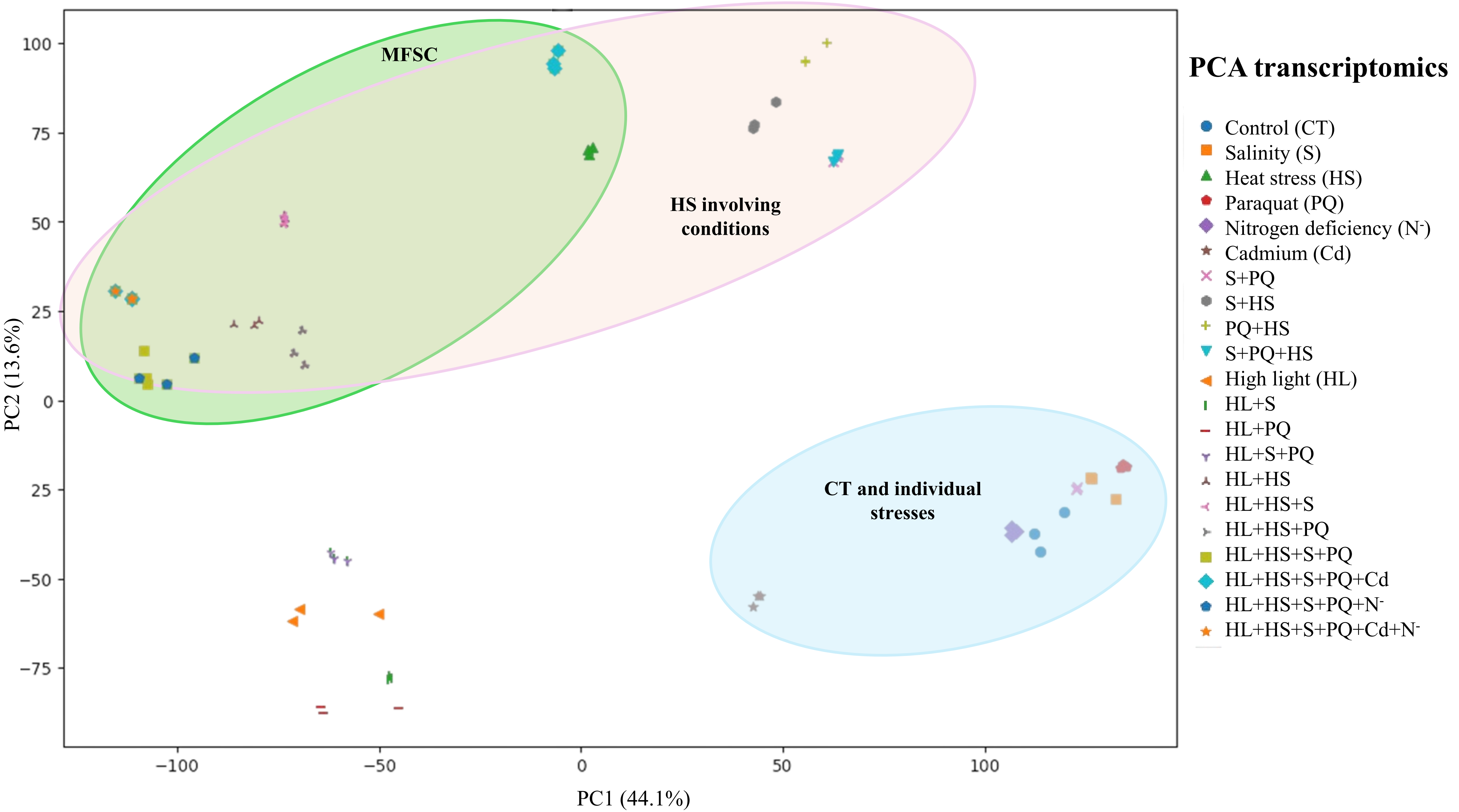

Supplementary Figure S1. Principal component analysis (PCA) showing differences in transcriptomic responses of *Solanum lycopersicum* to MFSC. Each point represents an individual biological replicate. Abbreviations: Cd, cadmium; HL, high light; HS, heat stress; N^-, nitrogen deficiency; PC, principal component; PQ, paraquat; S, salinity.

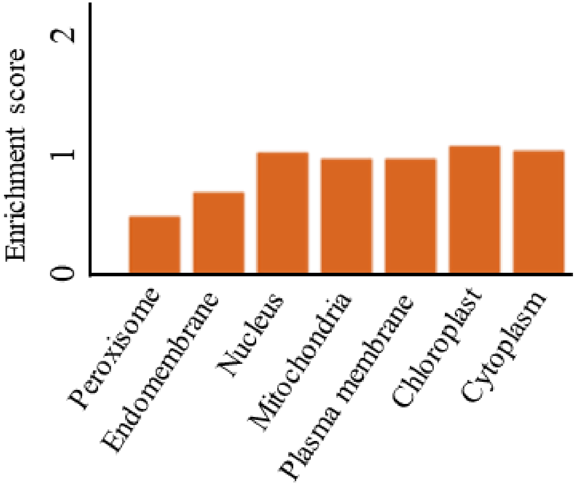

Supplementary Figure S2. Subcellular localization of MFSC-responsive transcripts. A gene ontology (GO) enrichment analysis of the Cellular Component category of differentially expressed transcripts common to 4, 5-, and 6-stress combinations in tomato (from Fig. 2A, common 6375 transcripts).

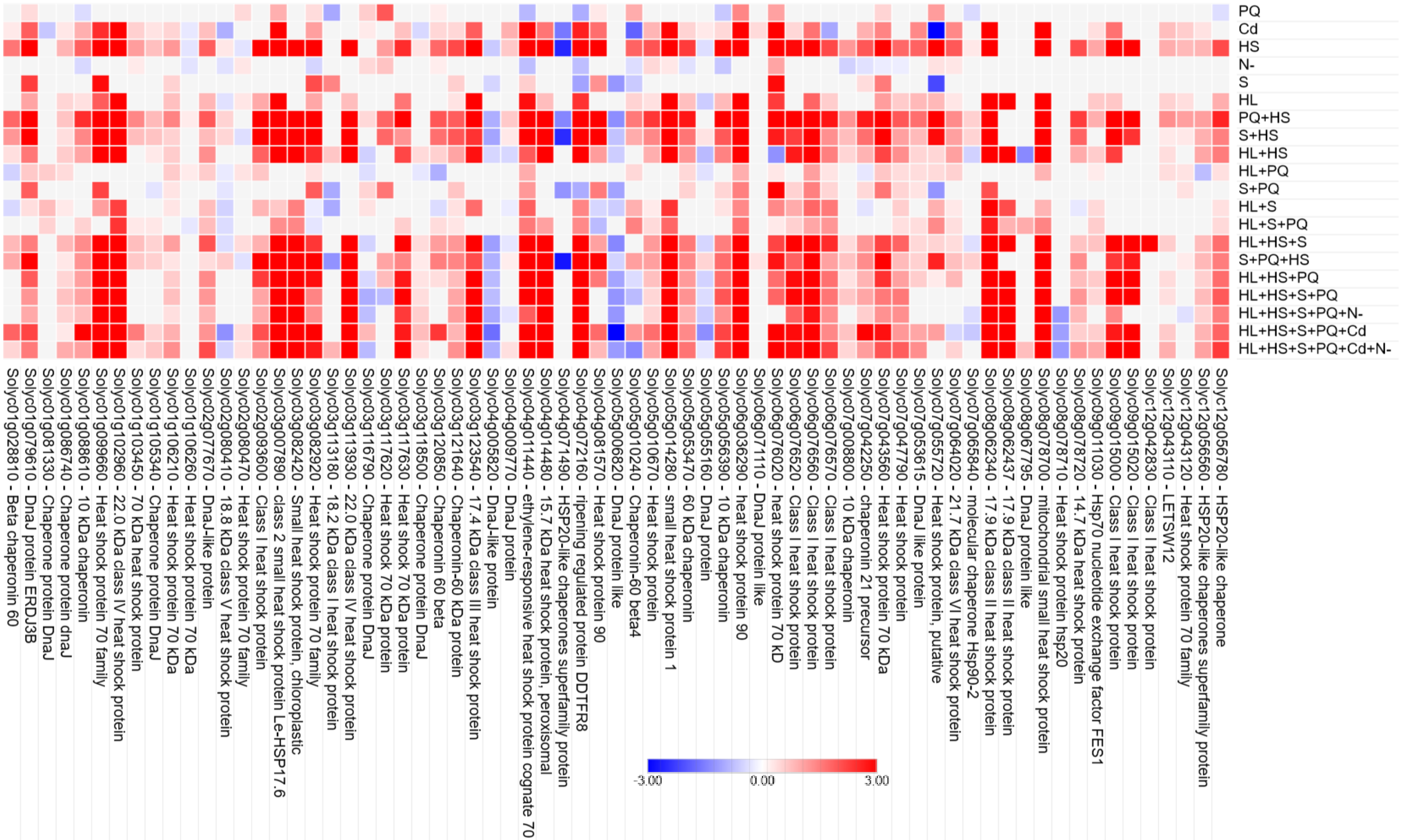

Supplementary Figure S3. Heatmap displaying the expression patterns of heat shock proteins (HSPs) across all stress conditions. Abbreviations: Cd, cadmium; HL, high light; HS, heat stress; N^-, nitrogen deficiency; PQ, paraquat; S, salinity.

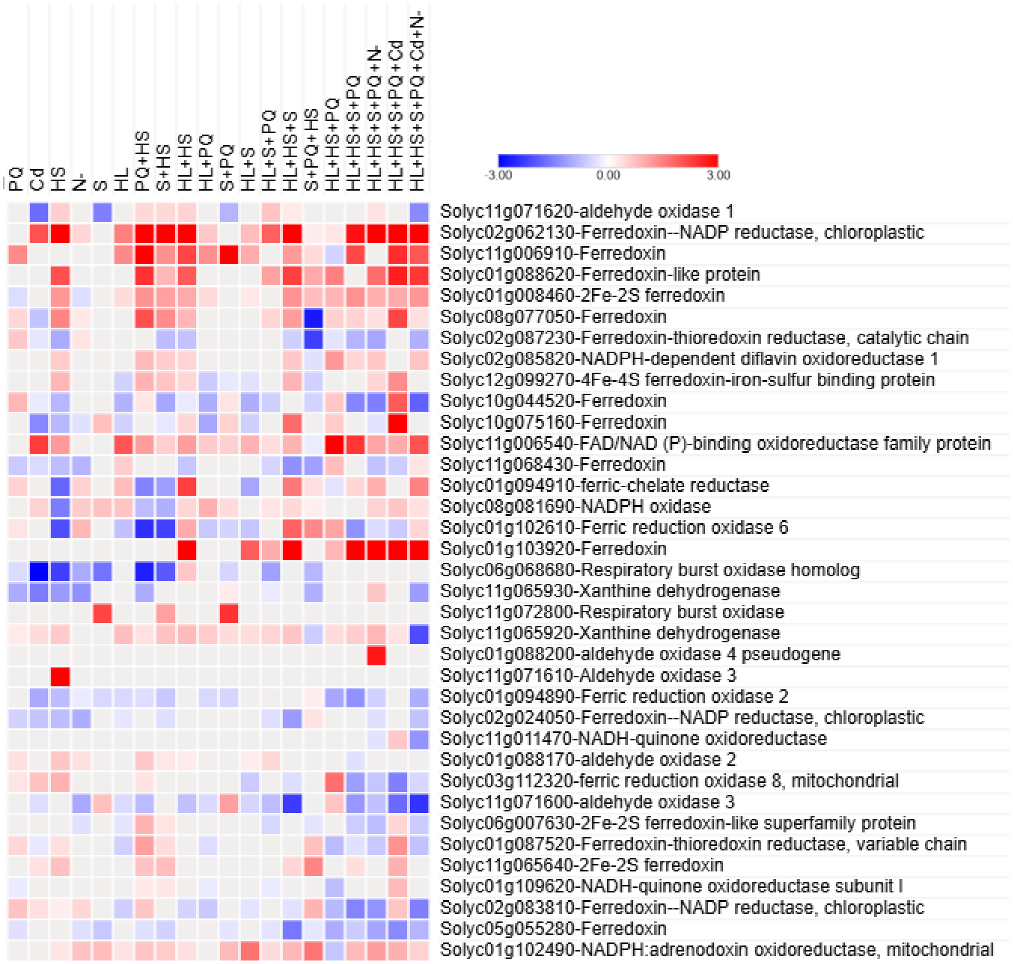

Supplementary Figure S4. Heatmap displaying the expression patterns of transcripts related to reactive oxygen species (ROS) production across all stress conditions. Abbreviations: Cd, cadmium; HL, high light; HS, heat stress; N^-, nitrogen deficiency; PQ, paraquat; S, salinity.

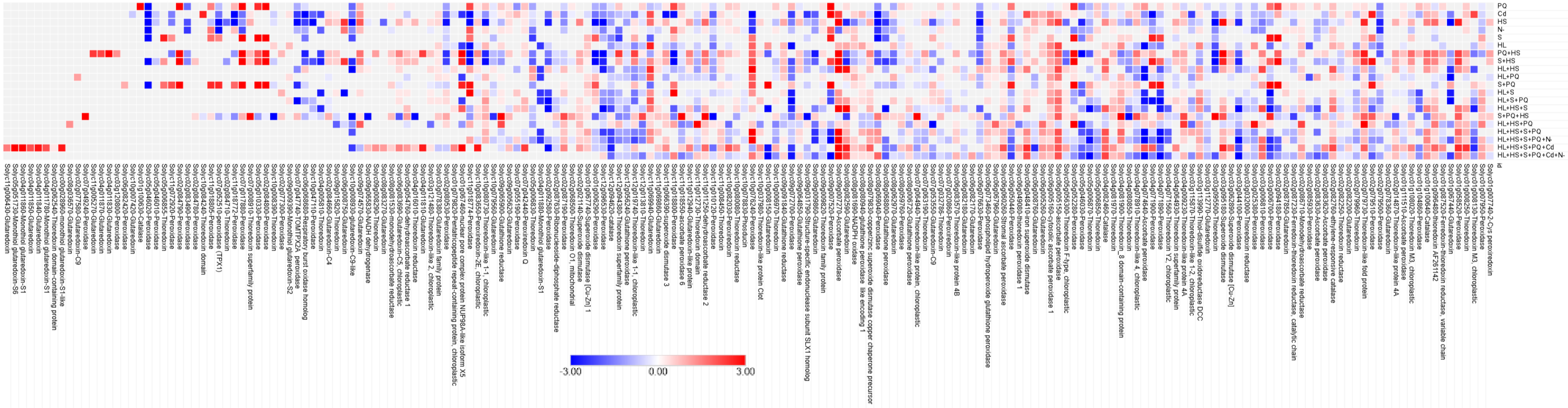

Supplementary Figure S5. Heatmap displaying the expression patterns of transcripts related to reactive oxygen species (ROS) scavenging across all stress conditions. Abbreviations: Cd, cadmium; HL, high light; HS, heat stress; N^-, nitrogen deficiency; PQ, paraquat; S, salinity.

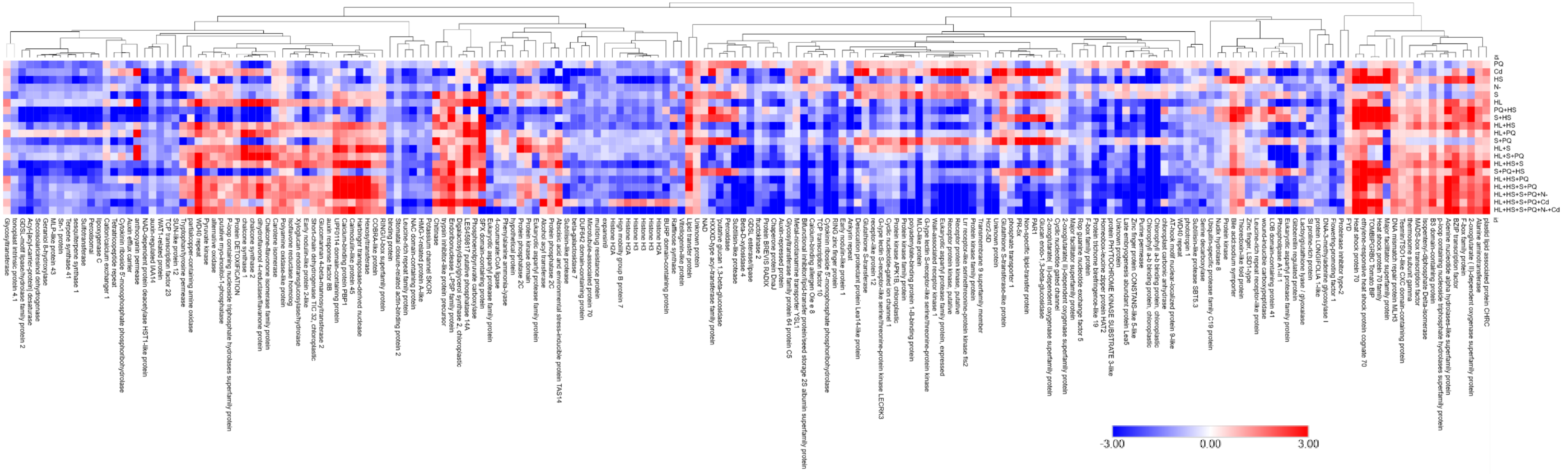

Supplementary Figure S6. Complete heatmap displaying the expression patterns of commonly altered transcripts across all stress conditions. Abbreviations: Cd, cadmium; HL, high light; HS, heat stress; N^-, nitrogen deficiency; PQ, paraquat; S, salinity.

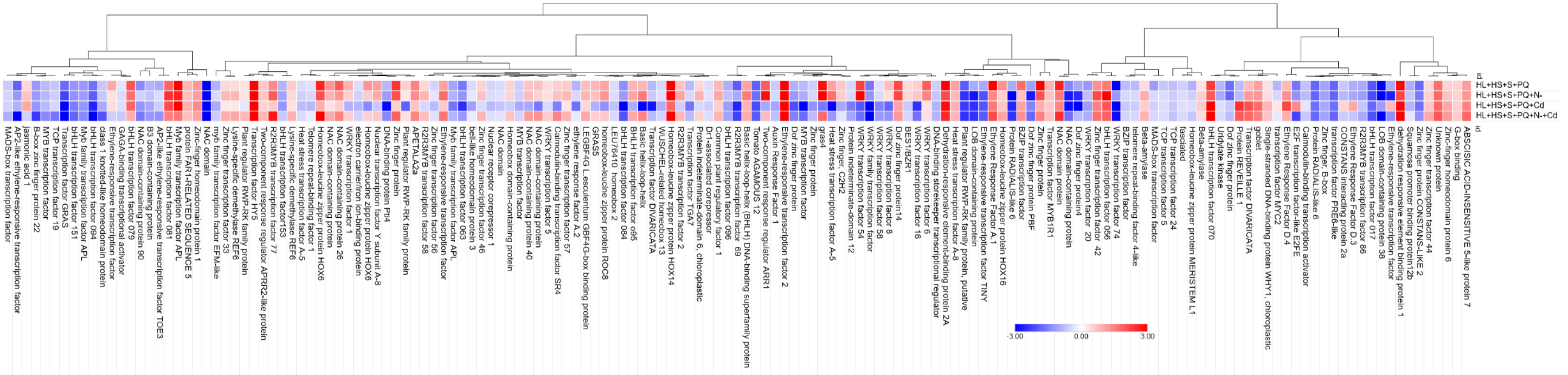

Supplementary Figure S7. Complete heatmap displaying the expression patterns of transcription factors specifically altered altered in response to 4-, 5-, or 6-stress combinations and not altered under other conditions. Abbreviations: Cd, cadmium; HL, high light; HS, heat stress; N^-, nitrogen deficiency; PQ, paraquat; S, salinity.

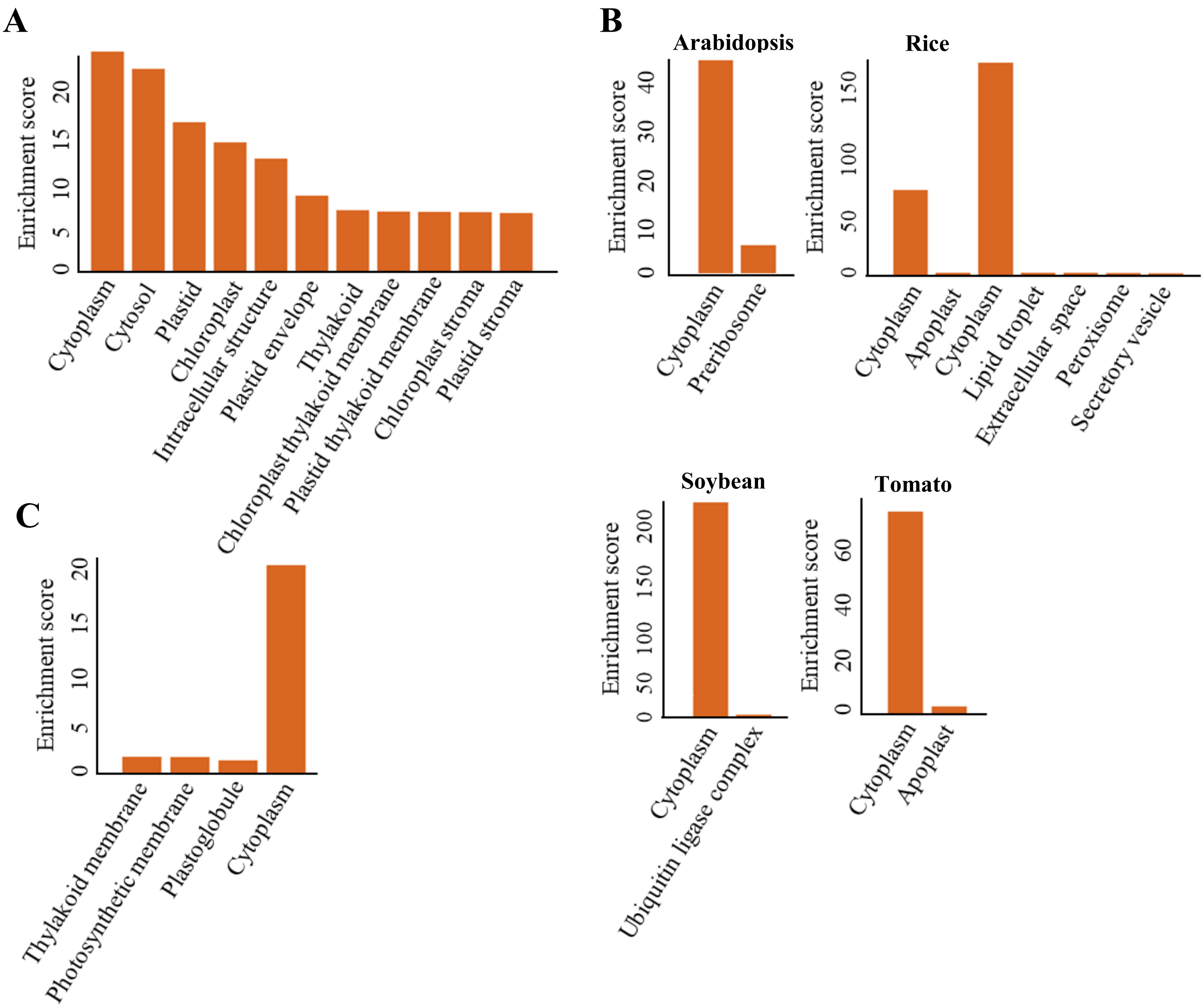

Supplementary Figure S8. Subcellular localization of MFSC-responsive transcripts in multicellular (tomato, soybean, rice, and Arabidopsis) and unicellular (*Chlamydomonas reinhardtii*) organisms. **A)** A gene ontology (GO) enrichment analysis of the Cellular Component category of the 213 genes commonly altered in tomato, Arabidopsis, soybean, and rice in response to MFSC (from Fig. 4A, B). **B)** A GO enrichment analysis of the Cellular Component category of transcripts specifically altered in each species under MFSC (2307 in Arabidopsis, 162 in rice, 6300 in soybean, and 727 in tomato; from Fig. 4A, C). **C)** A GO enrichment analysis of the Cellular Component category of the 117 genes common to the response of a unicellular (*Chlamydomonas reinhardtii*) and multicellular (tomato, Arabidopsis, soybean and rice) photosynthetic organisms to MFSC (from Fig. 5).

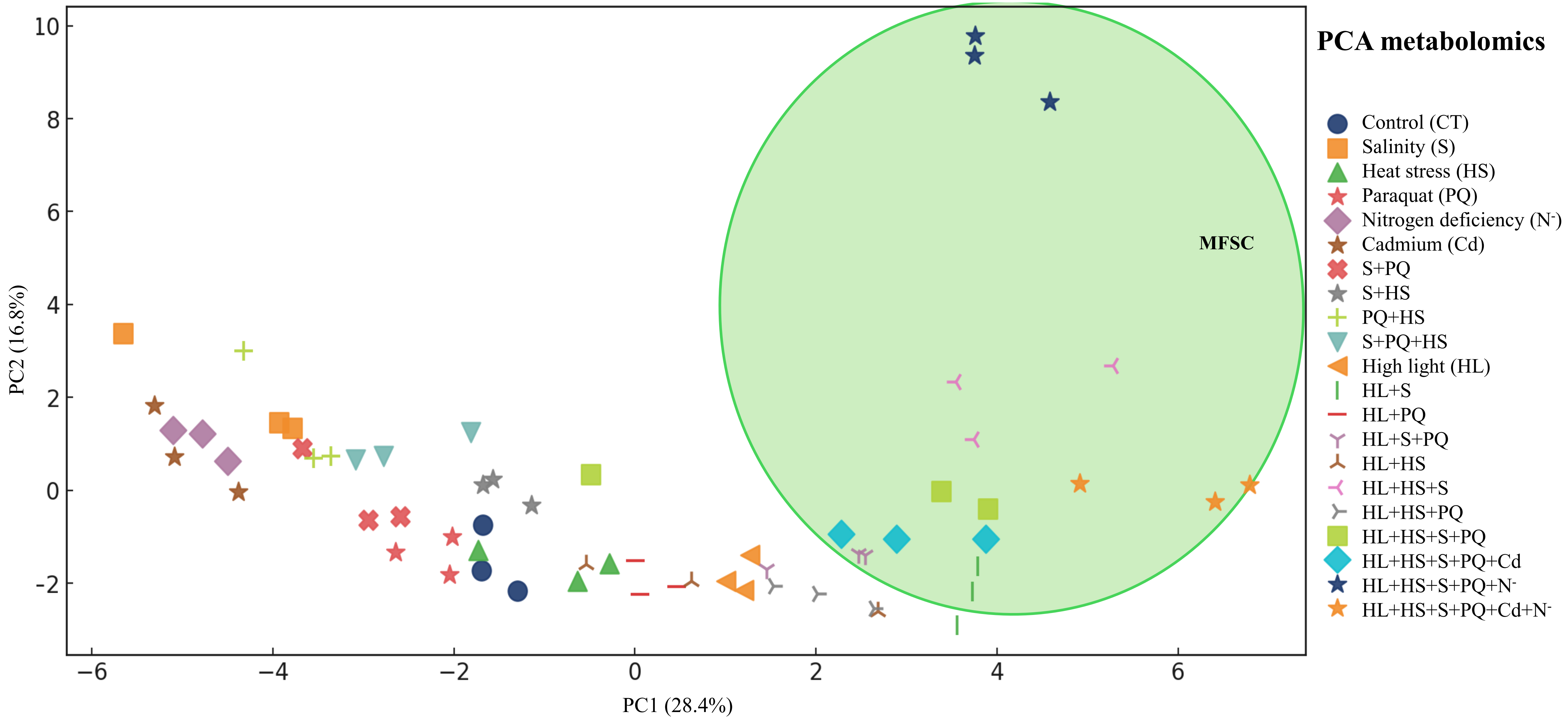

Supplementary Figure S9. Principal component analysis (PCA) showing differences in metabolomic responses of *Solanum lycopersicum* to MFSC. Each point represents an individual biological replicate. Abbreviations: Cd, cadmium; HL, high light; HS, heat stress; N^-, nitrogen deficiency; PC, principal component; PQ, paraquat; S, salinity.

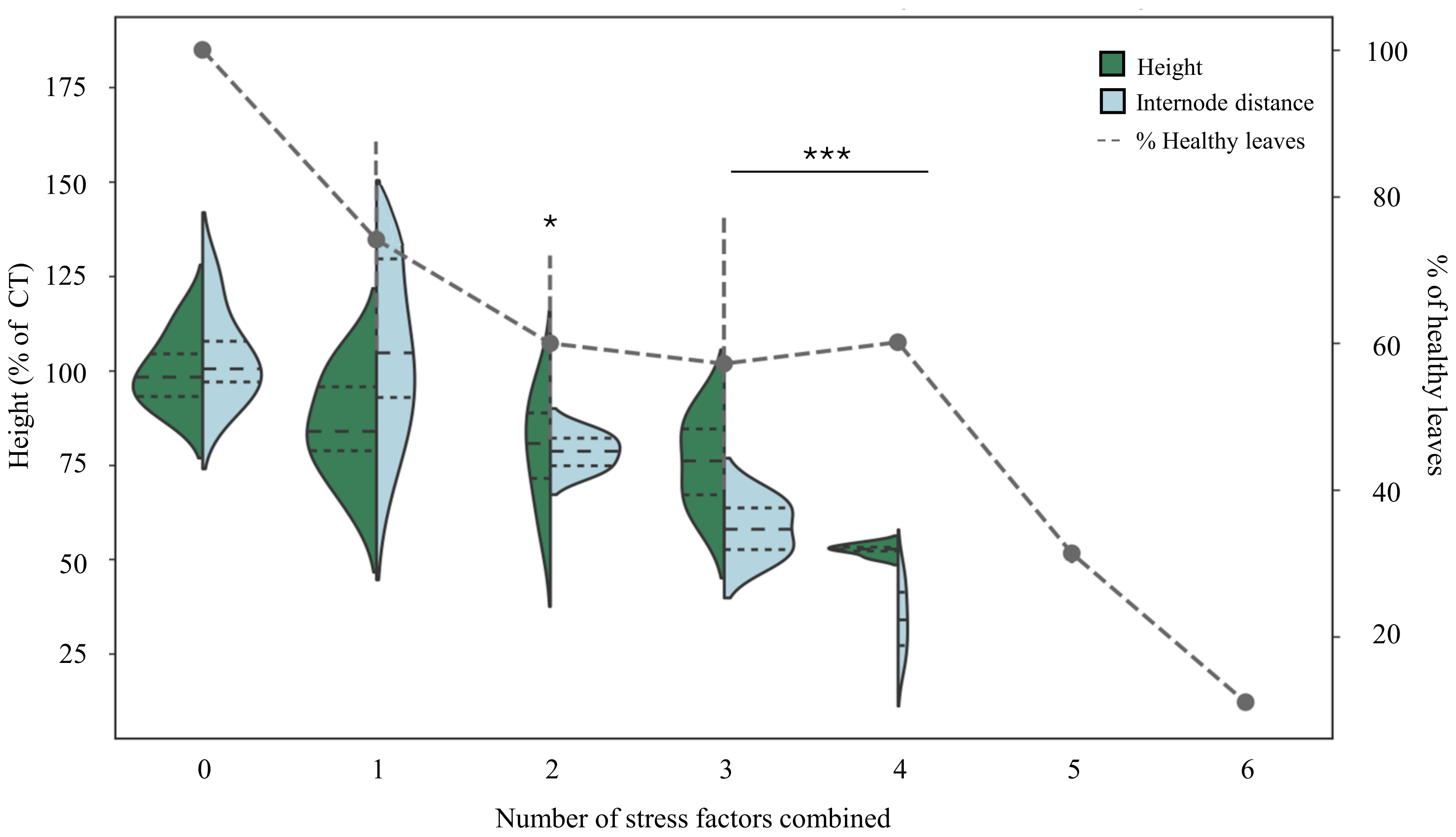

Supplementary Figure S10. Growth and morphological parameters in tomato plants exposed to multifactorial stress combination (MFSC). Raincloud plot showing the impact of increasing number of combined stress factors on plant growth and health. Violin plots represent the distribution of plant height (green) and internode distance (blue) for each stress combination level (0–4 factors; due to their short exposure time, HL and HS did not affect plant height and were therefore excluded from this measurement). Middle dashed lines within the violin plots represent the median, and lower and upper dashed lines within the violin plots correspond to the 25th and 75th percentiles of the data. Data are expressed as a percentage relative to control plants (CT). The dashed gray line represents the percentage of healthy leaves per plant across all stress combination (0–6 factors). Data extracted from Pascual et al. (2023).*$p < 0.05$; ***$p < 0.001$.
